# Data-driven computational modeling of CAR-T cell function

**DOI:** 10.3389/fimmu.2026.1707783

**Published:** 2026-05-13

**Authors:** Viren Shah, Justin A. Womack, Katie Palen, Bryon D. Johnson, Peiman Hematti, Tyce J. Kearl, Nirav N. Shah, Scott S. Terhune, Ranjan K. Dash

**Affiliations:** 1 Department of Biomedical Engineering, Medical College of Wisconsin, Milwaukee, WI, United States; 2 Blood and Marrow Transplant Program (BMT) and Cellular Therapy Program, Division of Hematology and Oncology, Medical College of Wisconsin, Milwaukee, WI, United States; 3 Cancer Center, Medical College of Wisconsin, Milwaukee, WI, United States; 4 Department of Microbiology and Immunology, Medical College of Wisconsin, Milwaukee, WI, United States; 5 Department of Physiology, Medical College of Wisconsin, Milwaukee, WI, United States

**Keywords:** CAR-T cell cytotoxicity kinetics, CAR-T cells, CAR-T cellular differentiation, CAR-T therapy, chimeric antigen receptor, computational modeling, mathematical modeling

## Abstract

**Introduction:**

The complex dynamics of chimeric antigen receptor T-cell (CAR-T cell) cytotoxicity and proliferation are potential factors that influence the clinical response to CAR-T therapy. The patient-specific functionality of CAR-T products play a role in these dynamics. CAR-T products comprise phenotypically and functionally distinct populations of cells that impact therapy response in different ways. We hypothesized that product-specific parameters exist that predict individual patient responses to therapy and that these can be elucidated by simulating the interactions of CAR-T products and tumor cells using an *in vitro* assay-based model.

**Methods:**

We use an ordinary differential equation (ODE)-based pharmacokinetic (PK) and pharmacodynamic (PD) model to characterize key CAR-T cell functional parameters. Parameters for the model developed using our method are product-specific and derived from *in vitro* assays performed on individual patient CAR-T products from clinical trial NCT04186520.

**Results:**

Our results demonstrate that while considerable variability is present in *in vitro* cytotoxicity kinetics and subsequently estimated model parameters between each product, these differences do not predict early (28 days) or late responses (90 days) after treatment across the total cohort of patients investigated. However, we show that differences in an estimated model parameter for increased CAR-T cell responsiveness to tumor cytotoxicity are correlated with durable therapy responses (no relapse through 180 days). Additionally, in a cohort of diffuse large B-cell lymphoma (DLBCL) patients, we demonstrate that a model parameter estimating cooperativity between CAR-T cells is also correlated with durable therapy responses and that may be related to differences in CD4:CD8 ratios in the CAR-T cell product.

**Conclusions:**

Overall, our work demonstrates that while pre-treatment CAR-T cell functional parameters vary on a patient and product basis, these parameters do not predict initial therapeutic responses. We find that initial therapeutic responses are possible across a range of initial product kinetic parameters. However, we observed that their potentially exist unique kinetic properties associated with the initial product that is predictive of disease relapse.

## Introduction

CAR-T cells are genetically modified T cells that express chimeric antigen receptors (CARs) recognizing tumor specific antigens to enable directed T-cell-mediated killing of tumor cells. CAR-T cell therapy has demonstrated efficacy in treating various B-cell malignancies (Reviewed in (1)). To date, the FDA has approved seven CAR-T products treating a wide range of hematologic malignancies. These include B-cell acute lymphocytic leukemia (BALL), chronic lymphocytic leukemia (CLL), diffuse large B-cell lymphomas (DLBCL), follicular lymphoma (FL), mantle cell lymphoma (MCL), and multiple myeloma (MM), with many more currently under development. However, despite the current success of the therapy, not all patients respond to treatment or sustain their initial responses. Thus, significant efforts are being directed toward developing methods to assess the functionality and differences of CAR-T products, which include both experimental (2) and computational approaches (3, 4).

The clinical response to CAR-T therapy is primarily governed by the kinetics of anti-tumor cytotoxicity, the proliferative capacity of individual patient-derived CAR-T products, and complexities of the tumor microenvironment (5, 6). Since each CAR-T cell product is unique to each patient, except in the case of allogeneic experimental CAR-T products under development, it is of great interest to develop a robust set of CAR-T-specific biomarkers. These would ideally be used to assess CAR-T products prior to therapy administration and correlate the CAR-T product characteristics with clinical responses to therapy. The primary challenge in developing reliable biomarkers is that the functionality of CAR-T products varies between patients based on a multitude of interrelated variables, each of which distinctly capable of modulating kinetics of therapeutic responses. Some of these variables include product-specific attributes such as CD4:CD8 cell ratios (7–9) and differentiation status of the CAR-T cells (10–12), which typically vary significantly between products (13). In addition, baseline interpatient variability exists that modulates product function, which is more difficult to assess (4). The overall effect of each variable modulating product functionality must be evaluated simultaneously to develop an accurate understanding of the key determinants involved in therapeutic response (14).

Assessments of CAR-T cells are typically completed prior to patient administration to ensure product viability, functionality (including CAR-T cell-mediated cytolysis and IFNγ expression when exposed to antigen-positive target cells), and immunophenotype (15). Data derived from these assays can also be used to elucidate the kinetics of anti-tumor cytotoxicity and proliferative capacities of CAR-T products using ordinary differential equation (ODE)-based pharmacokinetic (PK) and pharmacodynamic (PD) computational models (16, 17). However, to the best of our knowledge, *in vitro* computational models of CAR-T cells have been limited to describing the functional activity of cellular lines and have not been explicitly used to investigate the variability between patient-derived products used for therapy. Additionally, many computational frameworks exist for studying *in vivo* CAR-T therapy dynamics in treated patients (4, 6, 14, 18–24). These models, however, offer limited predictive value for future patients as model parameters derived from the kinetics of CAR-T cells post-infusion cannot be known for patients prior to therapy.

In this work, we utilize data from *in vitro* cytotoxicity assays performed on clinical CAR-T products for a cohort of 45 patients enrolled in clinical trial NCT04186520 (25). The trial investigates bispecific lentiviral CD20/CD19-targeted (LV20.19) CAR-T therapy in patients with relapsed, refractory (R/R) B-cell malignancies. We utilize cytotoxicity data in conjunction with an ODE-based computational model to characterize key functional parameters of CAR-T cells. The CAR construct used in this clinical trial contains 4-1BB/CD3ζ signaling domains along with tandem CD19 and CD20 binding domains that can target malignant B cells. Results from a cohort of patients in a separate study using the same CAR-T cell vector indicated that low *in vivo* effector-to-target (E:T) ratios impeded responses, and low *in vivo* expansion was associated with relapse (26). However, antigen loss of both CD19 and CD20 on the tumor was not found to be a route of therapeutic failure in that trial (25, 26). Using data gathered to date from this clinical trial, we tested the hypothesis that *in vitro*-derived model parameters, which define product cytotoxicity and proliferative capacity, vary between patient products and that these parameters can be used to predict clinical responses to therapy.

## Results

### Model parameterization and analysis

**Figure 1:**
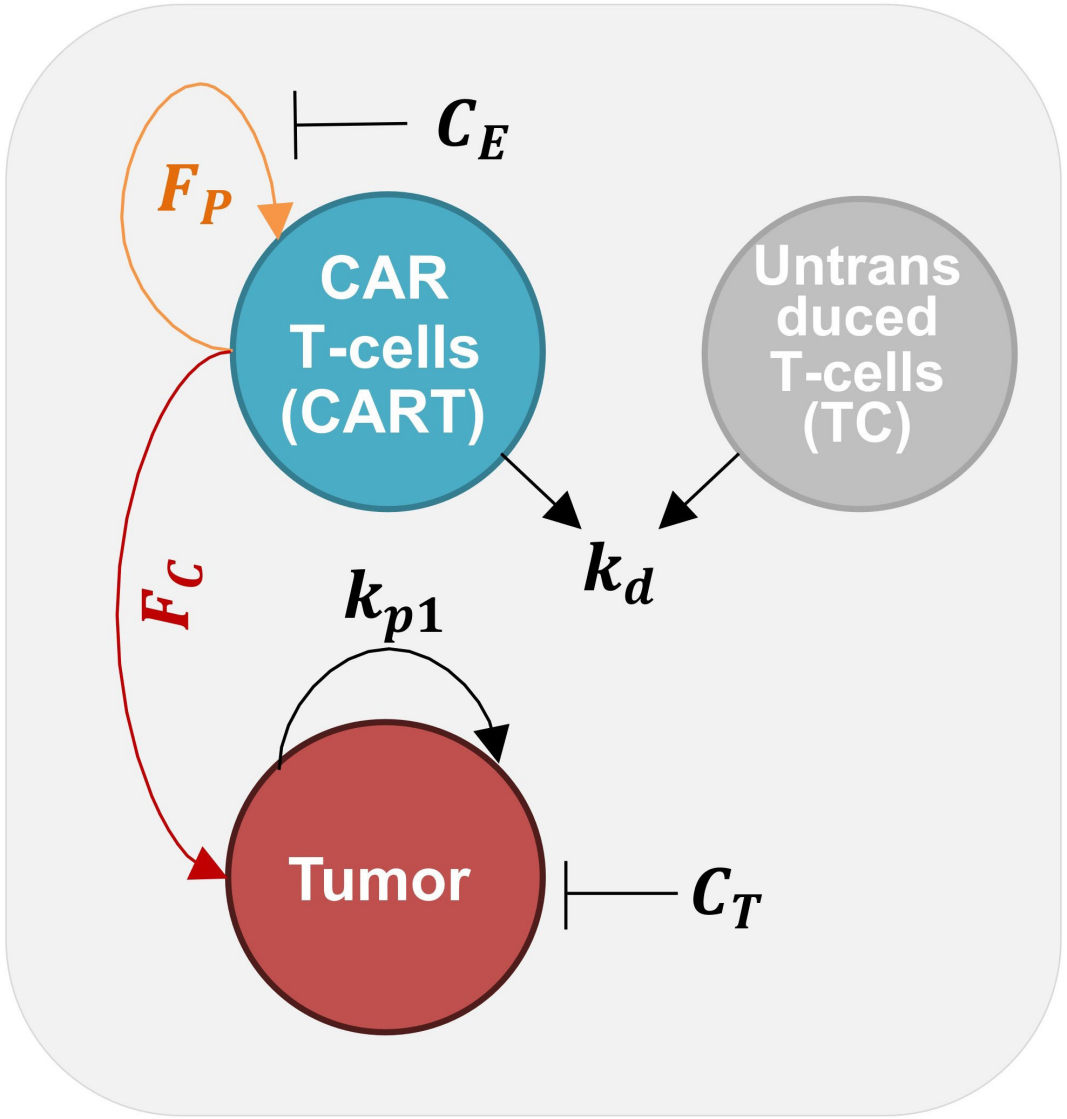
A three-compartment model for clinical *in vitro* CAR-T cell cytotoxicity assays. The model defines major interactions between tumor cells (T), CAR T-cells (CART), and untransduced T-cells (TC) populations in a controlled assay environment. Tumor cells are modeled to proliferate at a rate (k_p1_) restricted by an assay tumor carrying capacity (C_T_). CAR T-cell cytolysis of tumor cells is modeled by a function F_C_, and CAR T-cell proliferation response to tumor cytolysis by a function F_P_. CAR T-cell response is inhibited by an assay effector carrying capacity (C_E_). CAR T-cells and untransduced T-cells are modeled to decay independently of the other process at a rate k_d_.

**Figure 2:**
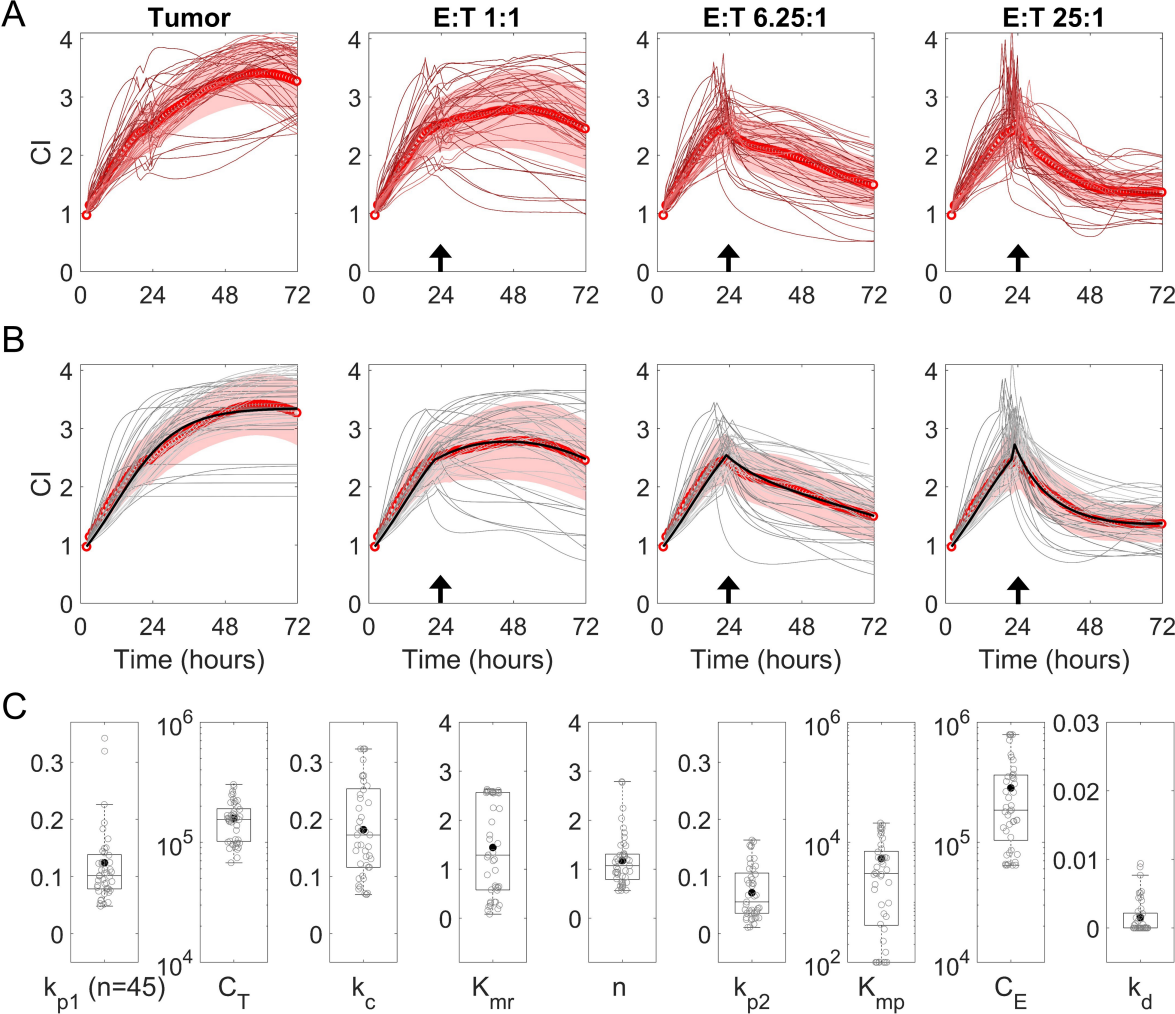
Analysis of relationships in bispecific LV20.19 CAR T-cell product cytotoxicities using an experimentally developed CAR T-cell cytotoxicity model. **(A)** Cytotoxicity assays of bispecific LV20.19 CAR T-cell against Raji cells, a B lymphoblastoid cell line, at various initial Effector (CART + TC):Target (Raji) (E:T) ratios, measuring cellular impedance (CI) over time. Effectors were added at 24 hrs noted by the arrow. (Red circles, mean CI; red shade, SD; red lines, individual patient assays). CI values per sample are scaled from 1 to 4. **(B)** Model simulation results for individual patient datasets and mean datasets from **(A)** Effectors were added at 24 hrs noted by the arrow. (Red circles, mean CI; red shade, SD; gray lines, individual model simulations, black line, mean model simulation) **(C)** Distribution of model parameter estimates across each patient dataset. Parameters are described in **Table 1**. Boxplots represent the median ± 25th percentiles, with whiskers representing the minimum and maximum values, excluding outliers (more than 2.7 standard deviations). Filled circle representing the mean. Deidentified patient data are from clinical trial NCT03019055 (n = 45).

To characterize the functionality of individual patient CAR-T products, we utilized the selected ODE model (**Figure 1**) (see Materials and Methods) to identify the best-fit functional parameters (**Table 1**) that described the cytotoxicity assay kinetics of each product (**Supplementary Figure 1**). Parameter sets were estimated for each product by fitting the model to normalized target control, target cytolysis, effector control, and co-culture assays. These data had previously been obtained for product prior to therapy administration. Tumor growth parameters k_p1_ and C_T_ were estimated using target control assays (**Supplementary Figure 2A**) by independently fitting the logistic growth term for targets in Equation 6. Effector decay parameter k_d_ was estimated using effector control assays (**Supplementary Figure 2B**) by independently fitting the exponential term used in Equations 7–8. This approach enabled us to independently account for inter-assay variability in control experiments and reduce the overall number of parameters required to be estimated using co-culture assays. Utilizing the parameter estimates for k_p1_, C_T_, and k_d_, the remaining model parameters, k_c_, K_mr_, n, k_p2_, K_mp_, and C_E_ were estimated using co-culture assays at varying initial E:T ratios (**Supplementary Figure 2C**). Individual patient target control assays, co-culture assays at varying E:T ratios, and average data are shown in **Figure 2A**, revealing unique trajectories across the entire patient cohort. Model simulations and parameter estimation for each patient assay were conducted in biological units (cells/time) rather than assay units of CI to ensure the portability of our methods and facilitate easier comparisons with other works. To enable this approach, conversions between assay units of CI and model units of cells were independently developed for each patient assay using linear calibration curves for targets (**Supplementary Figure 3A**) and effectors (**Supplementary Figure 3B**) based on known initial and final conditions. Individual model fittings to each patient target control and co-culture datasets are shown in **Figure 2B**. The individual model fittings are a result of a solitary estimated model parameter set, which minimized the unweighted sum-squared residuals (SSR) between estimated model CI and the observed CI from target control, co-culture, and effector control assays conducted at various initial E:T ratios in each patient dataset. The model fits using estimated parameter sets were assessed with R^2^ and SSR and generally resulted in good fits (90% of simulations with R^2^ > 0.80) and were also found to visually characterize the observed behavior between conditions and patients consistently. These parameters were selected from the results of 1000 Monte Carlo simulations with uniform randomized initial guesses within biologically relevant parameter upper and lower bounds, which were optimized using the fmincon algorithm in *MATLAB 2024b*. The distribution of these parameters across all patients is shown in **Figure 2C** and is found to vary considerably across the entire cohort of patient assays. The model upper and lower bounds used for parameter estimation, along with the calculated mean and standard deviation across individual parameter estimates, are shown in **Supplementary Table 1**. Overall, we identified best-fit parameter sets for each patient CAR-T product cytotoxicity assays and found considerable variation between the observed kinetics of the cytotoxicity assays and the resulting model parameter estimates across our patient cohort.

**Table 1:** Model parameters with associated biological definition and units.

Parameter	Biological definition	Unit
k_p1_	Tumor proliferation rate	1/hour
C_T_	Assay carrying capacity (tumor)	1/cells
k_c_	CAR-T cell cytolytic rate (saturated)	1/hour
K_mr_	CAR-T cell/tumor cytolytic constant (lower K_mr_ → transition from low to high rates at lower ratios)	unitless
n	CAR-T cell/tumor ratio exponent (higher n → steeper transition from low to high rates)	unitless
k_p2_	CAR-T cell proliferation rate (tumor driven)	1/hour
K_mp_	CAR-T cell proliferation constant (lower K_mp_ → transition from low to high rates at lower cytolysis)	cells/hour
C_E_	Assay carrying capacity (effectors)	cells
k_d_	CAR-T cell/T-cell death rate (basal)	1/hour

To further assess the overall model fit and sensitivity, an average dataset was developed by averaging all patient datasets (mean experimental dataset) and then parameterized using the same method as the individual patient datasets. Parameter estimates (mean data parameter estimate) for the mean experimental dataset are provided in **Supplementary Table 1**, which were found to be within one standard deviation of the mean across individual parameter estimates. A sufficiently high model goodness of fit was observed across all initial E:T ratios (R^2^ > 0.94) for the mean model dataset (**Supplementary Figure 4**). Error sensitivity analysis of effector model parameters was conducted to determine the model’s responsiveness to changes in estimates of these parameters. Assessing the absolute changes in the magnitude of model error with changes in parameter estimates is one aspect of understanding which model parameters are identifiable and not randomly assigned. Greater absolute changes in error demonstrate both increased parameter identifiability and significance in model results. Using the mean experimental dataset, it was shown that the model simulations are highly sensitive to effector cytotoxicity-associated model parameters, including k_c_, K_mr_, and n, while also demonstrating an acceptable level of sensitivity to effector proliferation parameters k_p2_, K_mp_, and C_E_. The model is shown not to be particularly sensitive to the effector decay rate, k_d_ (**Supplementary Figure 5A**), with the magnitude found to be notably lower than estimated cytotoxicity and proliferation rates across all assays. However, since this parameter can be estimated separately when the model is parameterized, it was considered identifiable and utilized for model simulations. Parameter correlation analysis of effector model parameters was also performed to assess model identifiability using the mean experimental dataset, demonstrating that model parameters estimated using the co-culture and effector control datasets are generally identifiable (**Supplementary Figure 5B**) while estimating in the parameter space of our model bounds (**Supplementary Table 1**). High correlations (> 0.80) were observed between model parameters C_E_ and n, and C_E_ and k_mp_, but given these were below the generally accepted cut-off for identifiability (0.93) they were considered identifiable for our analysis. Model simulations using mean data parameters with varying initial E:T ratios for predicted assay results in CI, % cytolysis of tumor cells, tumor cell population, effector cell population, CAR-T cell population, predicted untransduced T-cell populations, value of FC function, and value of FP function are provided in **Supplementary Figures 6A–H**. These simulations demonstrate a saturation of cytolysis rates in assays at approximately 12.5:1 E:T ratio, which can be helpful to account for in designing future assays. Additionally, below a E:T ratio of 1:2, cytolysis is observed to be negligible in the time frame of the cytotoxicity assay. A global sensitivity analysis was performed to compare changes in model parameters, initial assay E:T ratios, and assay cytolysis of tumor cells after 48 hours of co-culture (**Supplementary Figures 7A–G****).** Consistent with error sensitivity, these simulations demonstrate that changes in model parameters k_c_, K_mr_, and n have the most significant impact on modulating the achievable percentage of tumor cytolysis. Model parameters k_c_ and K_mr_ are, respectively, positively and negatively correlated with cytolysis with increasing values of the parameter and increasing initial E:T ratios. Model parameter n exhibits bimodal behavior in achievable cytolysis at higher values of n, with complete tumor cytolysis at higher E:T ratios and curtailed cytolysis at lower E:T ratios. This behavior dissipates at lower values of n, with simulations showing more consistent cytolysis but with lower overall magnitudes. Model parameter k_p2_ shows a slight positive correlation with increasing parameter values; however, this effect is curtailed at higher E:T as the carrying capacity of the assay is reached. Model parameters K_mp_ and k_d_ exhibit a slight negative correlation with cytolysis, while the carrying capacity has a slight positive correlation with increasing values and increasing initial E:T ratios.

### Relationship with clinical responses

**Figure 3:**
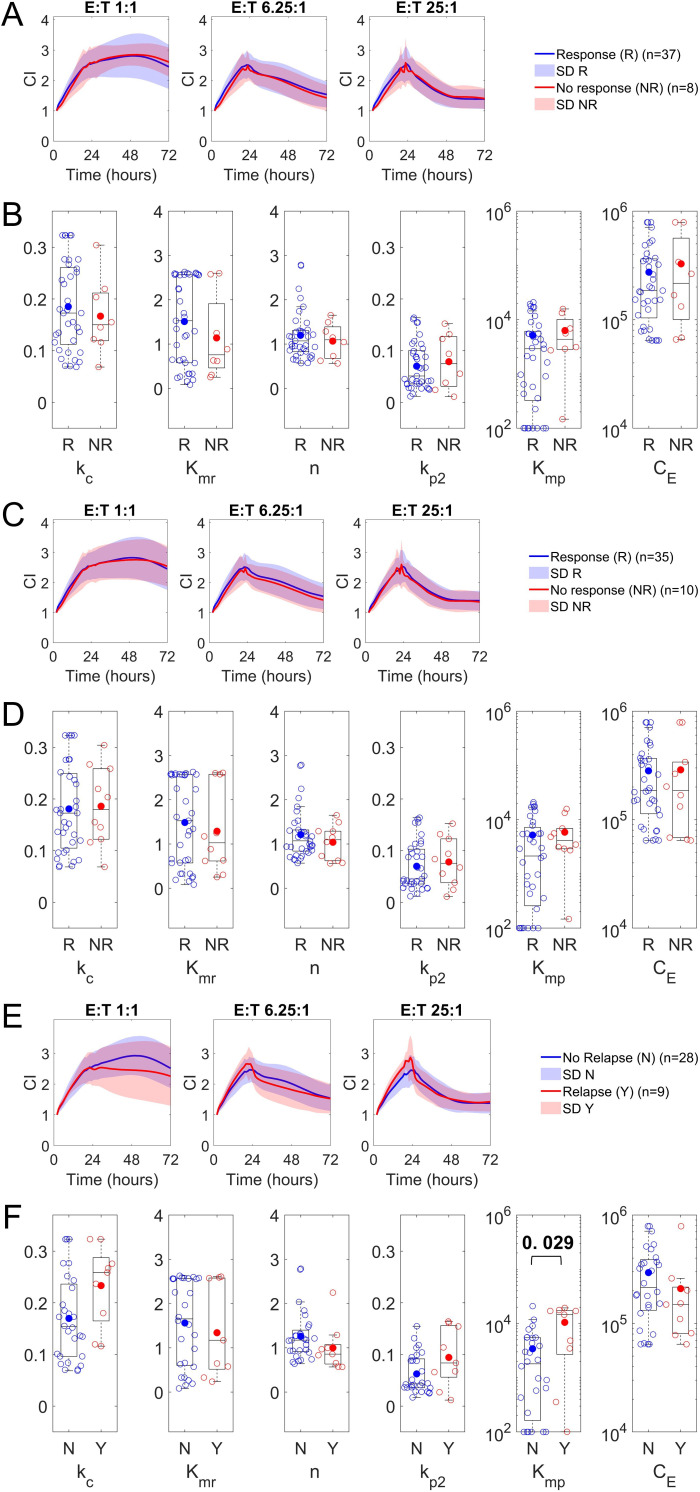
*In vitro* cytotoxicity data and model parameter distribution for therapy responders and non-responders at various time points. Model simulation results for individual patient datasets, comparing different time points and outcomes. **(A)** Day 28: Analysis of bispecific LV20.19 CAR T-cell cytotoxicity against CD19/20+ Raji cells at various initial Effector (CART + TC):Target (Raji) (E:T) ratios measured by cellular impedance (CI) over time and separated by therapy responders (R; blue) and non-responders (NR; red). Effectors were added at 24 hrs. **(B)** Distribution of model parameter estimates by day 28 response in the clinical dataset. **(C)** Day 90: Analysis of cytotoxicity by day 90 response in the clinical dataset. **(D)** Distribution of model parameter estimates by day 90 response in the clinical dataset. **(E)** Day 180 Relapse: Analysis of cytotoxicity by disease relapse status at 180 days. **(F)** Distribution of model parameter estimates by relapse (yes; red) or no release (no; blue) in the clinical dataset. R (responders, either complete response CR or partial response PR), NR (non-responder, i.e., progressive disease PD). Parameters are described in **Table 1**. Boxplots with a filled circle representing the mean. Statistical analysis of differences between parameter values among patient groupings was evaluated using the Wilcoxon rank sum test (significant P-values as shown, non-significant P-values of greater than 0.10 are not shown).

With the functional parameters of each clinical product having been established by fitting each patient assay to the selected model and observed to vary between patients, we next sought to understand if there existed any significant differences in parameter values among the subsequently observed clinical responses (**Supplementary Table 2**). All patient comparisons are after receiving LV20.19 CAR-T therapy with equal initial doses of 2.5 x 10^6^ CAR-T cells/kg. We omit further analysis of parameters k_p1_ and C_T_ as they are related to the tumor control and not associated with the patient product, and k_d_, given its negligible magnitude. Analysis of assay kinetics and parameter estimates by early therapy responses (day 28) (**Figures 3A, B**) and late therapy responses (day 90) (**Figures 3C, D**) demonstrate no differences between kinetics or among parameter estimates between patients with clinical complete or partial responses, which were grouped as therapy responders (R), and those with progressive disease, which are denoted as therapy non-responders (NR). Next, we analyzed parameters for patients in this cohort by disease relapse (through day 180) by removing therapy non-responders. We analyzed patients who had initial therapy responses (day 28 or day 90) and no relapse against patients with initial therapy responses and relapsed disease. Analysis of assay kinetics and parameters by disease relapse status (**Figures 3E, F**) reveals differences in CAR-T cell model parameter K_mp_ (p <0.05), which is the half-saturation constant for the CAR-T cell proliferation function, between patients without disease relapse (lower) and with disease relapse (higher). In the model, this parameter is associated with the responsiveness of CAR-T cells to proliferate after tumor cytolysis, and lower values indicate CAR-T cell populations that proliferate more quickly at lower magnitudes of the tumor cytolytic function (FC) and are thus more responsive. All p values are listed in **Supplementary Table 3**. However, it should be noted that the model has relatively low sensitivity to the parameter estimates (**Supplementary Figure 5A**), which may impact the accuracy of this observation. Additionally, we note differences in CAR-T cell cytotoxicity (model parameter k_c_), CAR-T cell cooperatively (model parameter n), and CAR-T cell proliferation rates (model parameter k_p2_); however, these were not statistically significant (p > 0.05). We also investigated whether model parameters correlated with patient side effects and found that patients who experienced CRS (cytokine release syndrome) had lower values of model parameter n (**Supplementary Figure 8A**); however, note the unequal sample sizes between the CRS and no CRS groups. We found no significant differences in model parameters between patients who experienced neurotoxicity after therapy or did not (**Supplementary Figure 8B**). Overall, these results indicate that while there is variability in parameter estimates between early and late R and NR groups, the average product kinetics and parameter estimates are similar. However, interestingly, we observed notable differences in the parameter estimates for patients who were able to maintain durable responses. This may indicate that while there are many possible parameter sets and kinetic trajectories that drive early and late therapy responses, durable long-term responses to CAR-T cell therapy (relapse status through day 180) are possibly associated with unique initial product kinetic properties.

Next, we investigated whether any properties associated with the product were associated with estimated model parameters. Since baseline differences exist between patient CD4:CD8 compositions and the differentiation status of CAR-T cells, and the manufacturing protocol can also alter the functionality of the CAR-T cell product, we were interested in understanding whether any of these differences affected CAR-T cell cytotoxicity assay kinetics across our patient cohort. All the products for LV20.19 CAR-T therapy were generated using a similar manufacturing protocol with IL-7/IL-15 cytokines; however, there were differences in the number of days the CAR-T cells were expanded before being assayed. We found no significant differences between manufacturing time and parameter estimates (**Supplementary Figure 9A**). This suggests that variations in manufacturing time did not significantly impact product functionality across the patient cohort. Additionally, changes in CD4:CD8 ratios (7, 27) and the differentiation status (10, 28–30) of the product are known to alter their functionality, with both lower CD4:CD8 ratios and lower terminally differentiated populations demonstrating increased tumor cytolysis. Model parameter n was observed to tend to decrease with increasing CD4:CD8 ratios across patient products, while other parameters displayed randomly scattered distributions (**Supplementary Figure 9B**). However, CD4:CD8 ratios in the total product were not found to be directly associated with therapy response or relapse in this patient cohort (**Supplementary Figures 9C, D, E**). No associations were found between model parameters and the cellular subpopulations of Tscm (stem cell memory), Tcm (central memory), Tem (effector memory), and Temra (terminally differentiated) cells contained in the product (**Supplementary Figures 10A–D**); however lower populations of CD4+ Temra (terminally differentiated) cells were correlated with response (**Supplementary Figures 11A–C**), but not relapse rate (**Supplementary Figures 12A–C**) across the overall patient cohort. This may indicate that the lack of product functionality resulting from increased CD4+ Temra is not reflected in the *in vitro* product assessment and model parameters and possibly manifests subsequent to therapy administration. Altogether, these results show that variabilities in the patient product manufacture and composition, with the possible exception of CD4:CD8 ratios, across the patient cohort do not translate to any apparent differences *in vitro* product parameters as predicted by our model.

**Figure 4:**
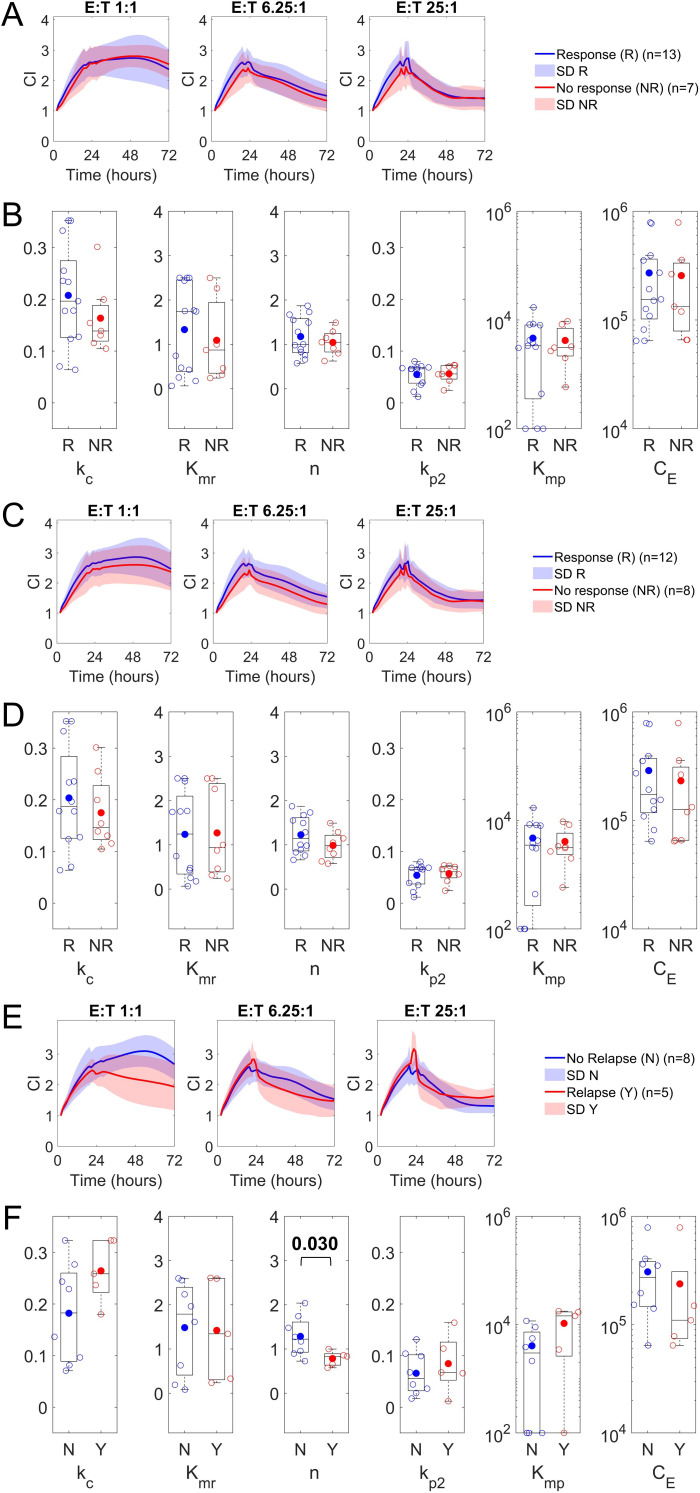
*In vitro* cytotoxicity data and model parameter distribution for therapy responders and non-responders for patients with DLBCL. **(A)** Analysis of bispecific LV20.19 CAR T-cell cytotoxicity against CD19/20+ Raji cells at various initial Effector (CART + TC):Target (Raji) (E:T) ratios measured by cellular impedance (CI) over time. Data were separated between therapy responders (R; blue) and non-responders (NR; red) at 28 days. (DLBCL, diffuse large B-cell lymphoma) **(B)** Distribution of model parameter estimates by day 28 responders (R) and non-responders (NR) in clinical dataset. **(C)** Analysis of cytotoxicity by day 90 in the clinical dataset. **(D)** Distribution of model parameter estimates by day 90 in the clinical dataset. **(E)** Analysis of cytotoxicity by disease relapse (Y; red) or no relapse (N; blue) status at 180 days. **(F)** Distribution of model parameter estimates by relapse in the clinical dataset. R (complete response CR or partial response PR), NR (i.e., progressive disease PD). Boxplots with a filled circle representing the mean. Statistical analysis of differences in parameter values among patient groupings was evaluated using a two-sided Wilcoxon rank sum test (significant P-values are shown; non-significant P-values of greater than 0.10 are not shown).

We also analyzed product kinetics in patients with different B-cell malignancies (DLBCL, MCL, FL, and CLL), as we hypothesized these may result in differences in the functionality of source patient T-cells used to generate the CAR-T cell product. We found notable variability in kinetics and parameter estimates between products depending on the patient malignancy type (**Supplementary Figures 13A, B**). Given that we observed differences between patient disease, and the fact that most of the NR day 28 (7/8), NR day 90 (8/12), and disease relapse (12/17) patients were from the DLBCL subpopulation, we next decided to analyze that cohort separately. Similarly, to the overall cohort of patients, we find no significant differences between R and NR for parameter estimates by day 28 response (**Figures 4A, B**) and by day 90 response (**Figures 4C, D**). Analysis by disease relapse status in this cohort reveals differences in the cytotoxicity assay kinetics between patients with relapse and those without relapse (**Figures 4E, F**). Comparison of parameter sets between these two groups shows that model parameter n (p < 0.05, ES = 0.50, CI [0.20, 0.86]), which was higher for patients with no disease relapse. Additionally, we noted differences in CAR-T cell cytotoxicity rates and proliferation rates between relapse status; however, these differences were not found to be significant (p > 0.05) (**Figures 4E, F**). We revisited the prior CD4:CD8 ratio analysis for the DLBCL cohort and observed similar trends for model parameter n with increasing CD4:CD8 ratios (**Supplementary Figure 14B**). CD4:CD8 ratios were not correlated with patient response or relapse status (**Supplementary Figures 14C–E**). Similar findings as the overall cohort were observed for product compositions as well (data not shown). In summary, these findings further support that early and late responses are possible with varying product kinetics; however, durable responses to therapy may result from unique initial product kinetic properties.

**Figure 5:**
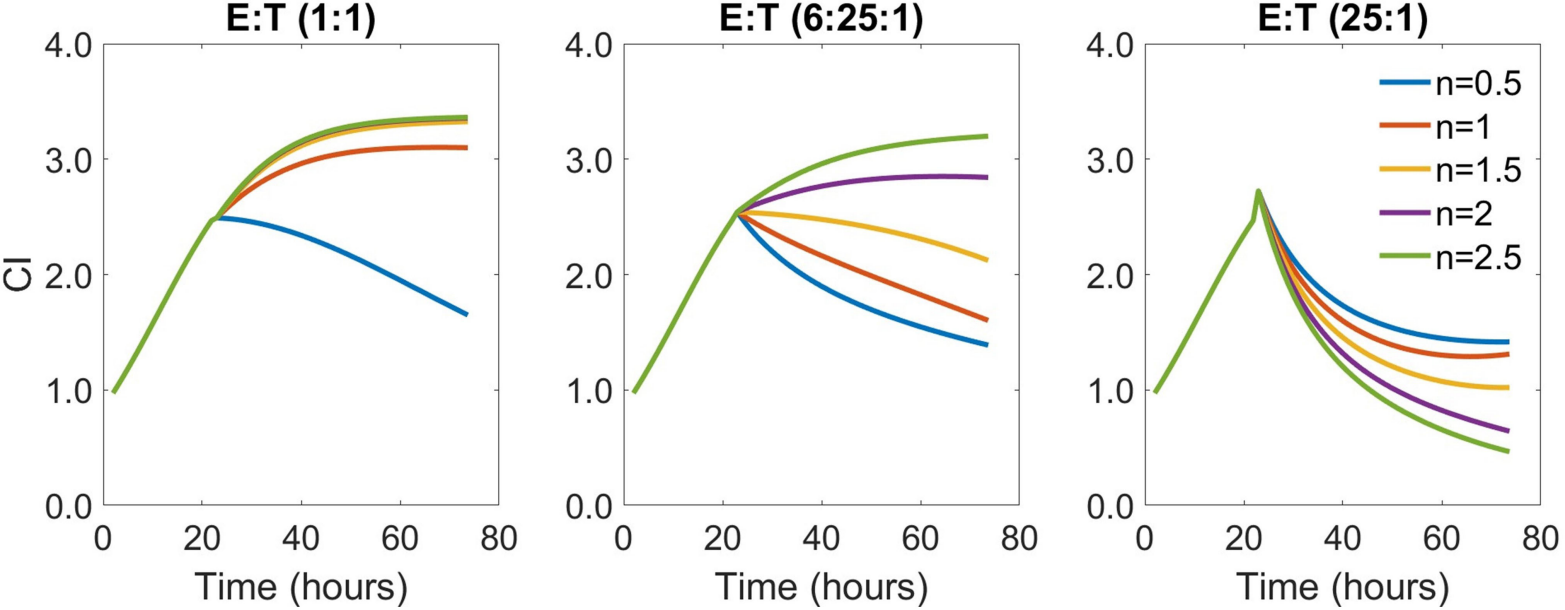
Model simulations that demonstrate how changes in model parameter n impact the trajectory of the predicted CI measurements from cytolysis experiments conducted across varying assay initial E:T ratios. Model parameter n is shown to increase the cytolysis of tumor cells by CAR-T cells (lower predicted CI) at higher values of n at high initial E:T ratios. However, higher values of n also result in decreased cytolysis of tumor cells (higher predicted CI) at lower initial E:T ratios. Simulations conducted using mean data parameter estimates while varying values of n from 0.5 to 2.

Since the most significant finding from the analysis of this patient sample was the differences in the estimates of model parameter n between DLBCL relapse and no-relapse patients, we simulated assay kinetics at varying values of n and varying initial E:T ratios (**Figure 5**). These simulations demonstrate that increasing n increases the overall depth of CI decrease or cytolysis achievable at higher initial E:T ratios. Still, increasing n is also observed to skew the system toward reduced cytolysis and increased tumor growth at lower initial E:T ratios. Given that these differences were only observed in the assay kinetics of relapse (lower n) compared to non-relapse (higher n), it may indicate that the ability of CAR-T products to increase the initial depth of the antitumor response when E:T ratios are higher *in vivo* may play a role in driving more sustained therapy responses.

**Figure 6:**
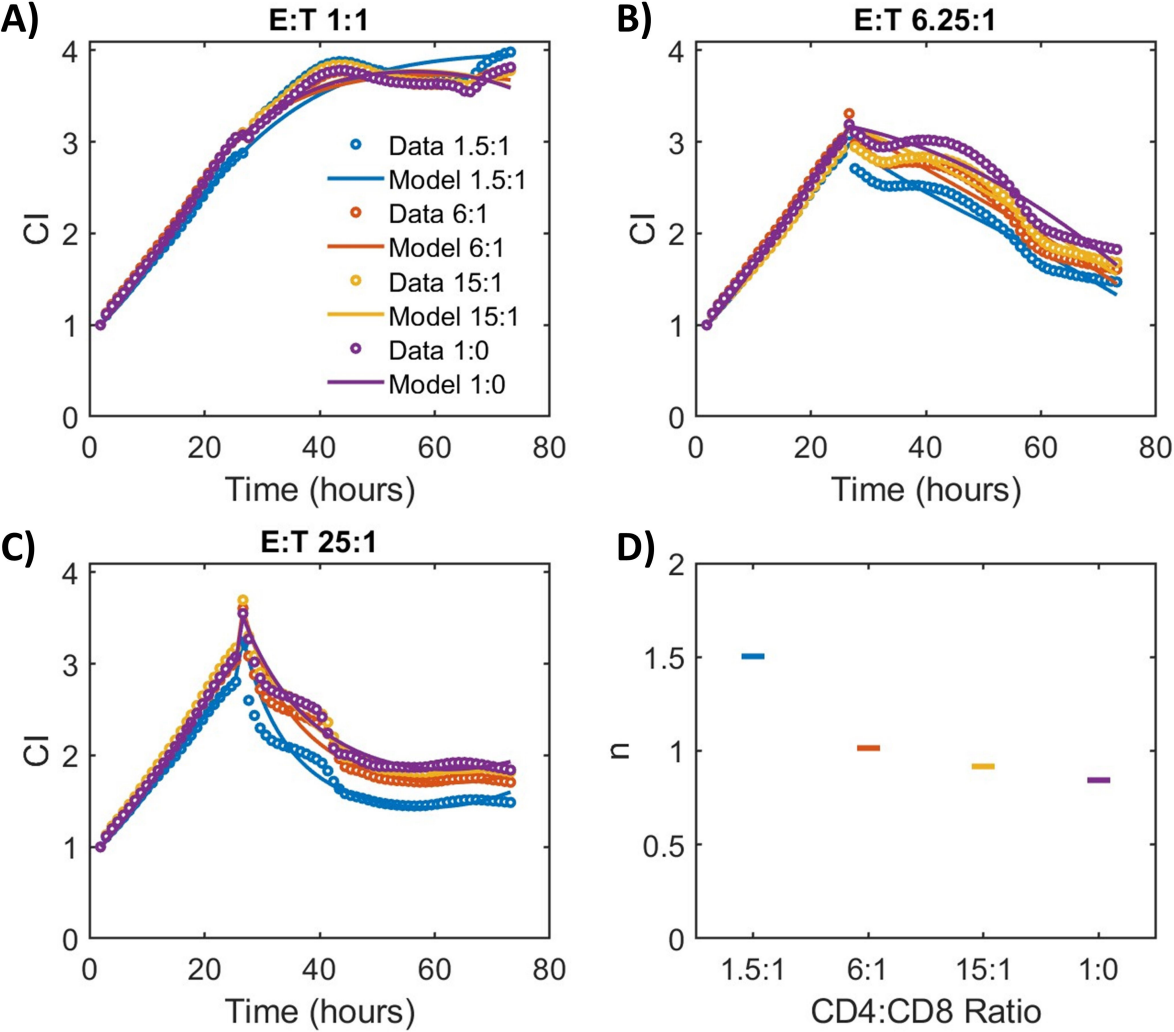
Cytotoxicity data and model simulations demonstrating how changes in CD4:CD8 ratios of a healthy PBMC donor-derived CAR-T product alter parameter **(n)** Analysis of bispecific LV20.19 CAR-T cell cytotoxicity against CD19/20+ Raji cells using CAR-T product manufactured with healthy PBMC donor, enriched for CD4 or CD8 cells, and adjusted to fixed CD4:CD8 ratios of 1:1, 4:1, 10:1 and 1:0 (CD4 only) among CD3+ cells which corresponded to 1.5:1, 6:1, 15:1, and 1:0 CD4:CD8 ratios (as denoted in legend) among CAR+ CD3+ cells, respectively. Various initial Effector (CAR-T cell + T cell):Target (Raji) (E:T) ratios measured by cellular impedance (CI) over time for **(A)** E:T 1:1, **(B)** E:T 6.25:1, and **(C)** E:T 25:1. **(D)** Predicted value for model parameter n at various CAR-T product CD4:CD8 ratios. Transduction efficiencies, model parameter estimates, and model goodness of fit are shown in **Supplementary Table 6**.

To test the hypothesis that lower CD4:CD8 ratios will increase the value of model parameter n and the maximum cytolysis achievable by the CAR-T product, we conducted cytotoxicity assays similar to those performed for the patient samples using a healthy donor PBMC-derived CAR-T product. The CD4:CD8 ratio of this singular CAR-T product was manipulated after manufacturing of the product by enriching for CD4 or CD8 cells. Cytotoxicity assays were then performed for the same CAR-T product at various initial E:T and CD4:CD8 ratios for a freshly manufactured CAR-T product (**Figures 6A–C****),** and also for the sample product after freezing and thawing cells for the assay (**Supplementary Figures 15A–C**). Model fitting and parameter estimation for the datasets were also performed following the same methods as developed for the patient CAR-T product datasets. The resulting parameter estimates for each CD4:CD8 ratio tested are shown in **Supplementary Tables 6**, **7**. The data demonstrate increasing absolute cytolysis (decrease in CI) with increasing E:T and decreasing CD4:CD8 ratios, and model simulations show a slight but notable decrease in estimated values for model parameter n with increasing CD4:CD8 ratios (**Figure 6D**). Additionally, we note that the overall TE for the product increased with increasing CD4:CD8 ratios, which rules out one confounding variable that may impact these findings and also demonstrates the increased potency of the lower CD4:CD8 ratio product compared to those with higher CD4:CD8 ratios, even with a lower number of CAR-T present in the assay. Altogether, these data show that changes in CD4:CD8 ratios following enrichment can influence the value of the model parameter n, which we had previously found to be correlated with CAR-T therapy relapse; however, CD4:CD8 product ratios do not fully or singularly control the value of n, which is consistent with the results from the analysis of the patient samples.

## Discussion

Using an ODE-based computational model, we characterized clinical CAR-T cell product-specific functional parameters based on *in vitro* cytotoxicity assays for each patient in a cohort of 45 patients receiving LV20.19 CAR-T therapy. We found that while these assay-derived model parameters demonstrated considerable interpatient variability, they were not correlated with early or late disease responses at days 28 or 90, respectively, across the entire cohort of patients analyzed. In patients with disease relapse before 180 days, we found that model parameter K_mp_ was positively correlated with relapse. Biologically, this parameter represents the responsiveness of CAR-T cell proliferation following tumor cytolysis, and lower values indicate a more effective product. Lower estimated values of this parameter in the initial patient product may facilitate higher CAR-T cell expansion *in vivo*, which in turn helps sustain therapeutic responses. This finding aligns with prior observations in a subset of patients from this study, which showed that high *in vivo* expansion was associated with absence of relapse (26). However, because of the broad variability in individual estimates for this parameter and poor sensitivity during model estimation, we do not believe it has the potential to be a reliable metric for predicting relapse. Other model parameters, such as maximum estimated rates of cytotoxicity and proliferation, varied across patients but did not show a significant correlation with relapse. We also find that parameter sets were not correlated with product differentiation status; however, we note a possible negative correlation between model parameter n and increasing CD4:CD8 ratios. Assay kinetics parameter sets were also observed to vary across the B-cell malignancy types present in this patient cohort. We hypothesize that these differences are potentially due to changes in the populations and functionality of patient-sourced T-cells associated with malignancy type (31, 32). While there is limited data comparing the responses of singular CAR-T cell therapies across multiple malignancies, as investigated in this dataset, therapy response also likely depends on the type of B-cell malignancy being treated (33). In fact, the *in vivo* product kinetics of CAR-T cells and functional model parameters necessary to achieve a response have been previously modeled and vary between malignancies (34). Altogether, this analysis and prior findings suggest that functional parameter sets aimed at predicting therapy responses will essentially need to be assessed for patient cohorts separated by malignancy.

In our data, given that most non-responders and relapsed patients were from the DLBCL cohort, we decided to assess parameter sets derived for these patients separately. DLBCL is generally associated with poorer prognosis and currently has high rates of disease progression after CAR-T therapy (35, 36). Here, we obtained similar results to those of the overall patient cohort, with our estimated parameter sets being uncorrelated with both early and late therapy responses. We did observe, however, that model parameter n was negatively correlated with disease relapse. This parameter can be understood to describe the cooperative tumor cell cytolysis of CAR-T cells within the population, and our findings indicate that CAR-T cell populations with higher levels of cell cooperativity may play a role in driving more durable responses. Biologically this parameter may be influenced positively by constructive intercellular interactions or impaired by suppressive interactions between various CAR-T cell phenotypes. Model simulations of parameter n using our mean patient dataset demonstrate that increasing the value of n increases the depth of cytolysis (the degree of CI drop) against tumor cells by CAR-T cells at high E:T ratios. Prior findings on a limited cohort of patients investigated in this study, as well as findings from other clinical studies, note that higher observed E:T ratios are strongly associated with durable therapeutic responses (3, 26). Based on this finding, the observed *in vivo* dynamics may be influenced by differences in parameter n, with higher values of n necessitating the need for achieving higher E:T ratios before effective CAR-T cell cytolysis of tumor cells and subsequently producing more durable responses. In the DLBCL cohort, we again observed that parameter n was negatively correlated with increasing CD4:CD8 ratios. This suggests that products with lower CD4:CD8 ratios may be more cooperative compared to those with higher CD4:CD8 ratios; however, significant interpatient variability was observed across this patient cohort, which prevents firm conclusions. To better understand the relationship between CD4:CD8 ratios and model parameter n and establish a biological basis for modulating this parameter, we performed *in vitro* cytotoxicity assays using a singular CAR-T product with varying CD4:CD8 ratios. Based on our studies and functional parameters derived from fitting the assay datasets to the computational model, we observed a negative relationship between increasing CD4:CD8 ratios and the model parameter n; however, this relationship was not particularly drastic across the wide range of ratios tested. This suggests that CD4:CD8 ratios can influence cooperatively but do not singularly control it, and it is possible that other subpopulations present in the CD4+ T cell population (such as regulatory FOXP3+ CD4+ T cells) may modulate this parameter more significantly and require further investigation in this context (37). Prior CAR-T therapy studies have demonstrated that lower CD4:CD8 ratios promote therapy responses (13, 27), although it is unclear if the response stems from the ratio or the difference in the absolute number of CD8+ CAR-T cells, which are expected to have higher anti-tumor cytotoxicity than CD4+ CAR-T cells (6). Manipulating product CD4:CD8 ratios has also been previously attempted for CAR-T cells, demonstrating that products with lower CD4:CD8 ratios exhibit higher *in vivo* cytotoxicity (7). One method for defining CD4:CD8 ratios in the clinical product involves the separate manufacturing of CD4+ and CD8+ CAR-T cell populations; however, this approach may result in hypo-functioning CD8+ CAR-T cells due to a lack of interaction with CD4+ T cells (38). Another possible method would be to alter the CD4:CD8 ratio using excess product available at the end of the manufacturing process; however, this leftover product will vary between patients and may not always be sufficient to lower the ratio significantly. Manufacturing products with defined CD4:CD8 ratios will likely present challenges that lead to increased complexity and cost of the manufacturing process, and it is unclear whether the CD4:CD8 ratio should be tailored to disease and patient characteristics. In our study, while this CD4:CD8 ratio was not statistically correlated with response or relapse in the overall patient cohort or DLBCL only cohort, we do note the presence of possible outliers in the dataset, without which the remaining patients without relapse were clustered around low CD4:CD8 ratios <5. In contrast, patients with relapsed disease status had CD4:CD8 ratios that skewed toward higher values.

Tumor antigen-loss, which is typically a significant aspect causing loss of responses, has not typically been observed in patients receiving LV20.19 CAR-T cells (39, 40). Yet durable therapy responses were not achieved for all patients in this cohort either. Our modeling results predict that intricate kinetics differences between how patient CAR-T cells initially proliferate and lyse tumor cells may influence these differences. However, one major limitation of our approach and the resulting findings is that product composition and function likely change once the product is administered to the patient, and it is possible that long-term product kinetics may not be similar to those which the model predicts for the initial product (12, 41, 42). This limitation is demonstrated by the fact that high CD4+ Temra populations were negatively correlated with initial therapy response in this dataset; however, they were not correlated with differences in *in vitro* assay-derived model parameters. It will be necessary to investigate longitudinal relationships for product kinetics further to fully understand how product functionality may change throughout the course of treatment. The modeling approach taken here will need to be extended to investigate how these *in vitro* model-derived parameters translate to observed *in vivo* therapy kinetics, not only clinical responses, to validate whether the pre-treatment assessment of product parameters can be a valid prognosticator.

Our assessment of each patient product separately using the computational model found that, for the most part, early and late therapy responses were possible across products displaying a wide range of cytotoxic and proliferative potential during *in vitro* assays. We believe that the absence of clear correlations between the cytotoxicity assay-derived functional parameters and the lack of initial responses to therapy is possibly associated with patient attributes and is not entirely dependent on product variability. The interaction of each patient’s product with their specific tumor cells was not assessed prior to therapy. Tumor cytolysis and CAR-T cell proliferation will likely depend not only on product-specific attributes but also on tumor-specific attributes, such as CAR-T cell affinity and avidity toward the tumor antigen or tumor cell-mediated immune suppression, which are more challenging to assess (43). Further refinement of assay methods, where the functionality of the CAR-T cell product is evaluated against the patient’s own tumor, may be warranted to better predict initial therapy failure. Additionally, the analysis done here was the result of varying product properties, which also included varying TE between patient products. The TE varied the number of CAR-T cells assayed across each patient assay. While we attempted to account for this in our computational model by changing the initial conditions based on TE, a more suitable method would be setting E:T ratios in the cytotoxicity assays based on CAR-T cells versus total T cells in the product since doses for infused product are fixed based on number of CAR-T cells and not total effectors as is evaluated in our data here. An assumption we made for our model was that all cytotoxicity observed in the assay was CAR-T cell mediated and T cells in the assay did not contribute. It would be important to validate this assumption with T cell: tumor control assays done in parallel with the co-culture assays and at similar ratios in the future. If this assumption is not valid then T cell mediated cytotoxicity would need to be included in the model and estimated separately. This change in how cytotoxicity is assessed between patient products could facilitate more comparable kinetic comparisons and potentially enhance the predictive value of *in vitro* product assessments prior to therapy. Overall, to produce pertinent model-based predictions, additional refinements in the types of assays and methods used to perform them will likely be needed to evaluate product functionality better and produce unbiased baseline comparisons between products.

Overall, our work demonstrates that while pre-treatment CAR-T cell functional parameters vary on a patient and product basis, these parameters, as estimated, are unlikely to predict initial therapeutic responses. We find that therapeutic responses are possible across a range of product kinetic parameters. However, we observed that there likely exist unique kinetic properties associated with the product that prevent disease relapse. As demonstrated by the DLBCL subgroup analysis, these parameters may be disease specific. There are likely additional variables, such as individual tumor characteristics and variables associated with the patient’s tumor microenvironment, that also modulate CAR-T cell kinetics and therapy responses. Further investigation into key disease and patient-specific variables and incorporating these variables into pre-treatment *in vitro* assays that assess product functionality will ultimately be necessary to develop a more accurate model prediction.

## Materials and methods

### Patient product samples and cytotoxicity assays

De-identified product information was provided by the MCW CAR T-cell Remnant Material Bank (IRB #PRO00042851) following the Medical College of Wisconsin IRB approval (IRB #PRO00049635). CAR-T products were manufactured as previously described from peripheral blood mononuclear cells (PBMCs) collected via apheresis of patients enrolled in NCT04186520 (25). Cytotoxicity assays were conducted on these samples as part of the correlative studies of the clinical trial. Briefly, target cells (Raji, a CD19+ and CD20+ lymphoma line) were cultured at 37 °C in RPMI (Roswell Park Memorial Institute) growth medium containing 10% FBS (fetal bovine serum), 1% L-glutamine, and 1% penicillin/streptomycin. The cells were washed and resuspended in fresh medium after 48 hr to ensure sufficient recovery, then adjusted to a concentration of 8 x 10^5^ cells/mL. The xCELLigence system was used for the cytotoxicity assays. Each well of a 96-well E-plate was coated with 4 µg/mL of anti-CD40 tethering reagent, incubated at room temperature for 3 hr, and then washed twice with PBS (phosphate-buffered saline). Next, 50 µL of growth medium at room temperature was added to each well before connecting the plates to the xCELLigence system, then incubating at 37 °C. A background impedance reading was taken after 30 min of incubation. The experiment was then paused, and Raji cells (targets) were added to wells at a density of 4 x 10^4^ cells/well. The plates were incubated at room temperature for 30 min to allow the cells to settle, then loaded onto the xCELLigence device. The experiment was resumed to monitor target cell attachment and proliferation, with impedance readings taken every 15 min for 24 hr. The xCELLigence instrument measurements are expressed as a cellular index (CI) value, which measures changes in electrical impedance in the assay. The addition and proliferation of cells causes changes in CI measurements, impeding electron flow through the assay (**Supplementary Figure 1A**). Effector cells were clinical lentiviral anti-CD20/CD19 (LV20.19) CAR-T products from different patients; the products included CAR-T cells and untransduced T-cells based on varying transduction efficiencies (TE), ranging from 9.8% to 48.8% (mean 24.87%, SD 8.75%). CAR-T cell clinical products were thawed, washed, and added to the xCelligence plates. These cells were loaded in triplicate wells at E:T ratios of 1:1 (4 x 10^4^ effectors), 6.25:1 (2.5 x 10^5^ effectors), and 25:1 (1 x 10^6^ effectors). Each well received 100 µL of effector cell suspension, bringing the total volume in each well to 200 µL. Triplicates of target-only and effector-only wells were included as negative controls, and wells receiving cytolysis reagent served as positive controls. The E-plates were then returned to the xCELLigence system, and data acquisition resumed. A total of 289 readings were taken to collect the impedance of each well, after which the plates were removed. Target cells, which are adherent, and effector cells in suspension, distinctly alter the total CI measurements obtained from the assay (**Supplementary Figures 1B, C**).

### Cytotoxicity assays with varied CD4:CD8 ratios

To study the effect of varying CD4:CD8 ratios, we used a CAR T product that was generated from PBMCs of a normal healthy donor. After manufacturing, the CAR-T cell product TE, overall CD4 and CD8 composition, and CAR-expressing CD4 and CD8 composition were assessed using flow cytometry (**Supplementary Table 6**). CAR-T products were enriched by immunomagnetic selection for either CD4 cells or CD8 cells using Miltenyi microbeads, and cytotoxicity assays were performed on a single xCELLigence plate for four overall CD4:CD8 ratio manipulated products (1:1, 4:1, 10:1, 1:0) along with the original products versus Raji lymphoma target cells. The CAR-T product was subsequently frozen and then thawed for further analysis. After thaw, the CAR-T product TE, overall CD4 and CD8 composition, and CAR-T cell CD4 and CD8 composition were assessed using flow cytometry (**Supplementary Table 7**), and cytotoxicity assays for CD4:CD8 ratio manipulated product (1:1, 4:1, 10:1, 1:0) were conducted similarly to that described above.

### Patient cytotoxicity and clinical datasets

Since the cytotoxicity data gathered from the xCELLigence system varied in terms of starting CI and overall magnitude, we chose to normalize patient datasets prior to parameterizing our computational model. Patient target control, effector control, and co-culture datasets collected from xCELLigence cytotoxicity assays were normalized collectively for each patient using Equation 1 to ensure a magnitude between 0 and 4 CI.

(1) $$CI_{Norm}=(\frac{\text{CI }-\;CI_{Min}}{CI_{Max}-\;CI_{Min}})*4$$

We used the time point after target addition in the data as time = 0 hours and utilized this as our initial time point for the target control assays during model parameter estimation and derivation of calibration curves. We derived the time point for effector addition, which was approximately 24 hours in the cytotoxicity datasets, by using the time point after the instrument was recorded as returned to the platform in the xCELLigence message log. We used this time point as our initial effector addition time point for the co-culture and effector control assays during parameter estimation and calibration curve derivation. The dataset was also shifted to a fixed starting point of 1 CI after target addition (time = 0) for better visualization and comparison. To develop an average dataset (mean experimental dataset), we combined all datasets by using the target control dataset for the first 24 hours of data and the co-culture datasets starting from the individual experiment’s initial effector addition timepoints for the next 48 hours of data.

Additionally, the patient data contained the clinical response for each patient who received LV20.19 CAR-T therapy. Each patient received a dose of 2.5 x 10^6^ CAR-T cells/kg with a maximum total dose of 2 x 10^8^ CAR-T cells. The distribution of patient responses after receiving therapy, along with the categories used for model comparisons, is summarized in **Supplementary Table 2**.

### Varied CD4:CD8 ratio cytotoxicity datasets

The cytotoxicity datasets for varied CD4:CD8 ratios were normalized individually across the collective target control, effector control, co-culture data for each ratio tested (1:1, 4:1, 10:1, 1:0) and starting points shifted, similar to the process followed for the individual patient datasets, which is described above. Additionally, the CI data for the time point of effector addition was calculated by summing the CI from the prior time point (before effector addition) and the CI after effector addition from the corresponding effector control. This was done to account for the timepoint missed between plate removal, effector addition, and plate return, which corresponds to the initial conditions for the model after effector addition.

### Model selection

We aimed to select a mechanistic mathematical model that could characterize the cytotoxicity and proliferation of CAR-T cells across the range of initial effector-to-target (E:T) ratios and cell numbers used in our cytotoxicity assays, while concurrently reducing the number of parameters needed to represent the system properly. We started with commonly used models of ecological systems based on predator-prey dynamics. These have been previously used to model both *in vivo* and *in vitro* CAR-T cell dynamics (16, 24). In these models, tumor growth is modeled as logistic growth with a growth rate (k_p1_) limited by a carrying capacity (C_T_) (44). CAR-T cell cytotoxicity (FC) against tumor cells occurs at a cytotoxicity rate (k_c_) proportional to both the population of tumor cells (T) and CAR-T cells (CART). A proliferation rate (k_p2_) proportional to both T and CART represents the CAR-T cell proliferation response (FP) to the tumor (16, 45). CAR-T cells are modeled to decay at a rate (k_d_) proportional to their population (16, 45). The general system is represented by Equations 2–5.

(2) $$\frac{dT}{dt}=k_{p1}*T*(1-\frac{T}{C_{T}})-F_{C}$$

(3) $$\frac{dCART}{dt}=F_{P}-\;k_{d}*CART$$

(4) $$F_{C}=k_{c}*CART*T$$

(5) $$F_{P}=k_{p2}*CART*T$$

However, we observed that these models are unable to accurately describe the cytotoxicity (FC) and proliferation (FP) of CAR-T cells across varied initial E:T ratios of CAR-T cells and tumor cells with a single parameter set and would require reparameterization at each E:T ratio to produce accurate parameter estimates.

We instead chose terms for FC and FP based on previously derived ratio-dependent models for *in vivo* T-cell dynamics, which have also been used to model CAR-T therapy responses *in vivo* (46). The general system is represented in **Figure 1** and by **Equations 6**–**10**. The parameters used in the model, along with relevant biological descriptions, are presented in **Table 1**.

(6) $$\frac{dT}{dt}=k_{p1}*T*(1-\frac{T}{C_{T}})-F_{C}$$

(7) $$\frac{dCART}{dt}=F_{P}*ln(\frac{TC+CART}{C_{E}})\;-\;k_{d}*CART$$

(8) $$\frac{dTC}{dt}=-\;k_{d}*TC$$

(9) $$F_{C}=k_{c}*\frac{{\left(CART/T\right)}^{n}}{K_{mr}^{n}+{\left(CART/T\right)}^{n}}*T$$

(10) $$F_{P}=k_{p2}*\frac{{\left(F_{C}\right)}^{2\;}}{K_{mp}^{2}+{\left(F_{C}\right)}^{2\;}}*CART$$

In this model, the FC function is modeled as a Hill-type function, where the kc is modulated by the ratio of tumor cells to CAR-T cells. The ratio term used in this equation is analogous to drug concentrations in the standard Hill equations. This function introduces two additional parameters into the model, K_mr_ and n. Model parameter K_mr_ determines the half-saturation of the ratio for k_c_ with increasing values of K_mr_ representing a reduction in the ability of the CAR-T cell product to eliminate tumor cells at lower E:T ratios. Model parameter n represents the cooperativity between CAR-T cells in the product population. Increasing n values translate to CAR-T products, which are more effective at eliminating tumors at higher E:T ratios and have significantly curtailed tumor elimination at lower E:T ratios. We also chose to include a logarithmic term, based on the Gompertz growth equation, to modulate and restrict CAR-T cell responses toward a carrying capacity, as used in previous *in vivo* CAR-T therapy models (21, 22). Based on observations in effector control assays (**Supplementary Figure 2B**), we chose to omit any tumor-independent growth for CAR-T cells. We included untransduced T-cell populations (TC) as the effector cells in our patient assays, which included both CAR-T cell and T-cell populations. This varied depending on the TE during CAR-T cell manufacturing for each patient. To avoid introducing further parameters, we assume that TC does not have any anti-tumor activity in this assay as they do not recognize antigen domains present in Raji cells utilized in the assay and decays at the same rate as CAR-T cells in this assay, given that they are from the same biological source.

### Statistical analysis

To assess the statistical significance of differences between model parameter estimates and product compositions between different patient groups (**Supplementary Table 1****),** a non-parametric two-sided Wilcoxon rank sum test (α = 0.05) was utilized. Strict multiple testing corrections were not applied to avoid high Type II error rates.

## Data Availability

The original contributions presented in the study are included in the article/**Supplementary Material**. Further inquiries can be directed to the corresponding author.
