# Supplementary material for: Data-driven computational modeling of CAR-T cell function
Source: Front Immunol. 2026 May 13;17:1707783. doi: 10.3389/fimmu.2026.1707783 (PMC13212496; doi:10.3389/fimmu.2026.1707783)
Supplement: Supplementary file 1 [file Presentation1.pdf]

Supplementary Figures and Tables

Data-Driven Computational Modeling of CAR-T Cell Function

Viren Shah[1], Justin A. Womack[1], Katie Palen[2], Bryon Johnson[2,3], Peiman Hematti[2,3], Tyce J. Kearl[2,3], Nirav N. Shah[2,3], Scott S. Terhune[1,3,4*,] and Ranjan K. Dash[1,3,5*]

[1]Department of Biomedical Engineering, Medical College of Wisconsin, Milwaukee, WI 53226

[2]Blood and Marrow Transplant Program (BMT) and Cellular Therapy Program at the Medical College of Wisconsin, Division of Hematology and Oncology, Medical College of Wisconsin, Milwaukee, WI 53226

[3]Cancer Center, Medical College of Wisconsin, Milwaukee, WI 53226

[4]Department of Microbiology and Immunology, Medical College of Wisconsin, Milwaukee, WI 53226

[5]Department of Physiology, Medical College of Wisconsin, Milwaukee, WI 53226

*Equal contributions

Corresponding Author: Ranjan Dash

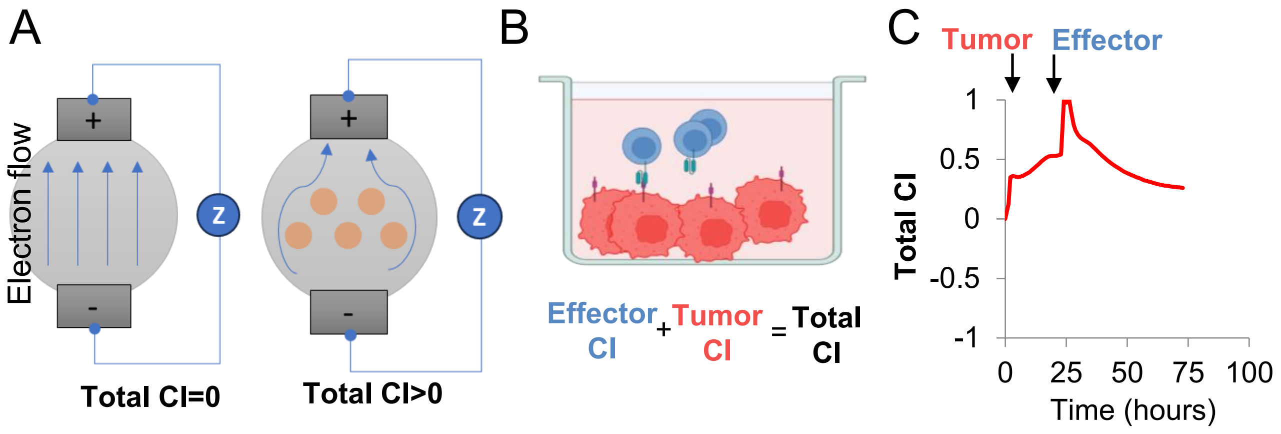

Supplemental Figure 1. xCELLigence cytotoxicity assay schematic. (A) The assay measures electrical impedance through culture media with a Cell Index (CI) = 0 representing free electron flow and CI > 0 impeded electron flow. Addition of cells to the culture impedes electron flow. (**B)** Assay CI measurements are a combination of adherent tumor cells (CD19+ Raji cells) and non-adherent effector cells (CAR-T cells and untransduced T-cells). **(C)** Sample co-culture assays are completed by addition of tumor cells for ~24 hrs allowing proliferation and then addition of effector cells at ~24 hrs, monitoring for an additional 48 hrs. CI values are obtained in real-time for the duration of the assay with T cell expansion and tumor killing reflected in the change in impedance and CI over time.

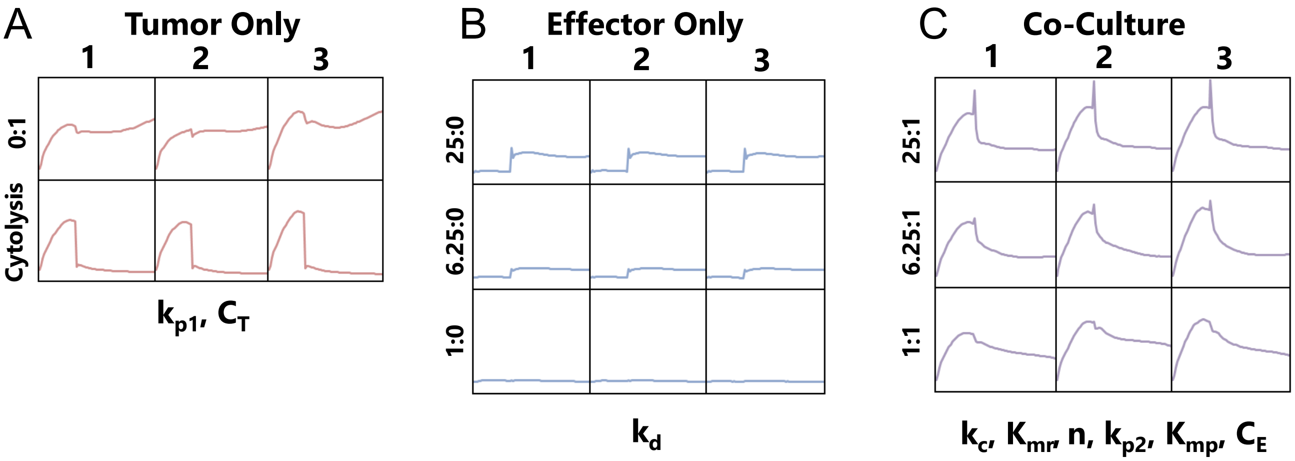

Supplemental Figure 2. Conditions used for parameter estimation. (A) Tumor cell only (E:T at 0:1) and induced cytolysis assays were used to estimate k_{p1} and C_T, respectively. **(B)** Effector only conditions (E:T at 25:0, 6.25:0, 1:0) starting at 24 hrs were used to estimate k_d. **(C)** Tumor and effector co-culture conditions at varying E:T ratios (25:1, 6.25:1, 1:1) with tumor cultured for 24 hrs followed by addition of effectors. Co-culture conditions were used to estimate model parameters k_c, K_{mr}, n, k_{p2}, K_{mp}, C_E. Parameters are defined in Table 1. Data are from a representative patient sample (Patient 11). n=3

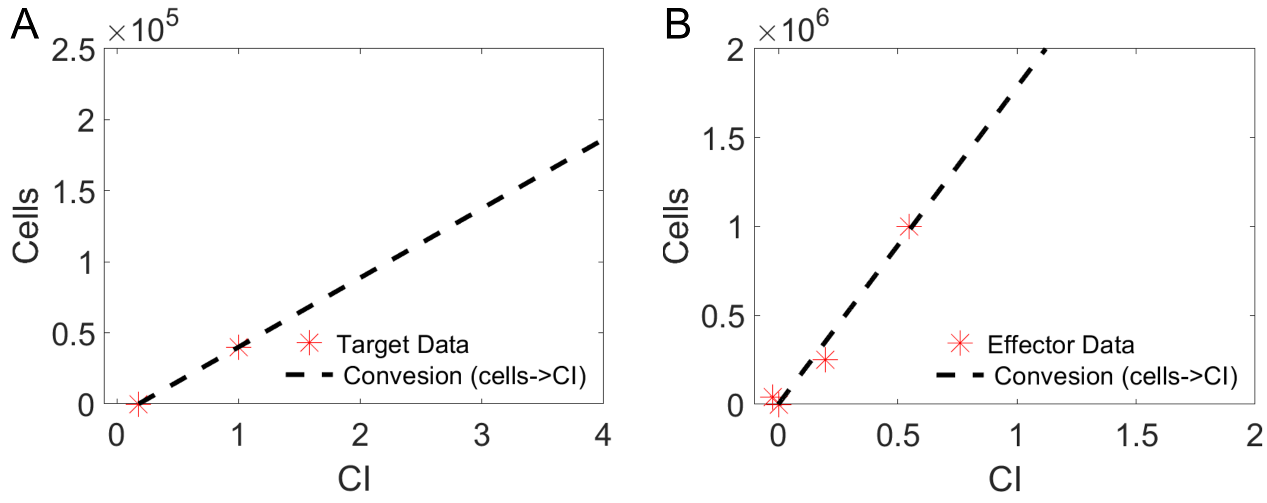

Supplemental Figure 3. Calibrations curves for converting between CI and cells. (A) Conversion between target CI and target cells using the initial CI measurement (t=0 hours) of tumor cell only data and final CI measurement of tumor cell cytolysis data (t=72 hours) at a E:T ratio of 0:1. **(B)** Conversion between effector CI and effector cells using the initial CI measurement of effector cell only data (t = 24 hours) at varying initial E:T ratios of 1:0, 6:25:0, and 25:0. A 1:1 E:T ratio represents a cell number of 40,000 effectors to 40,000 targets. Data are from a representative patient sample (Patient 11). n=3.

Supplemental Table 1. Model parameters bounds, mean of all individual parameter estimates for all patient datasets (mean), parameter estimate using mean patient data (mean data parameter estimate)

Parameter	Model bounds	Mean	Mean standard deviation	Mean data parameter estimate
$\mathbf{k_{p1}}$	[0, 0.40]	0.12	0.08	0.11
$\mathbf{C_T}$	[4e4, 3e5]	1.53e5	5.87e4	1.27e5
$\mathbf{k_c}$	[0, 0.40]	0.18	0.08	0.12
$\mathbf{K_{mr}}$	[0, 4]	1.44	0.96	1.16
**n**	[0, 4]	1.16	0.52	0.80
$\mathbf{k_{p2}}$	[0, 0.20]	0.07	0.05	0.04
$\mathbf{K_{mp}}$	[1e2, 4e4]	5.52e3	6.14e3	2.30e3
$\mathbf{C_E}$	[9e4, 6e5]	2.83e5	2.32e5	4.49e5
$\mathbf{k_d}$	[0, 0.02]	1.50e-3	2.50e-3	3.20e-3

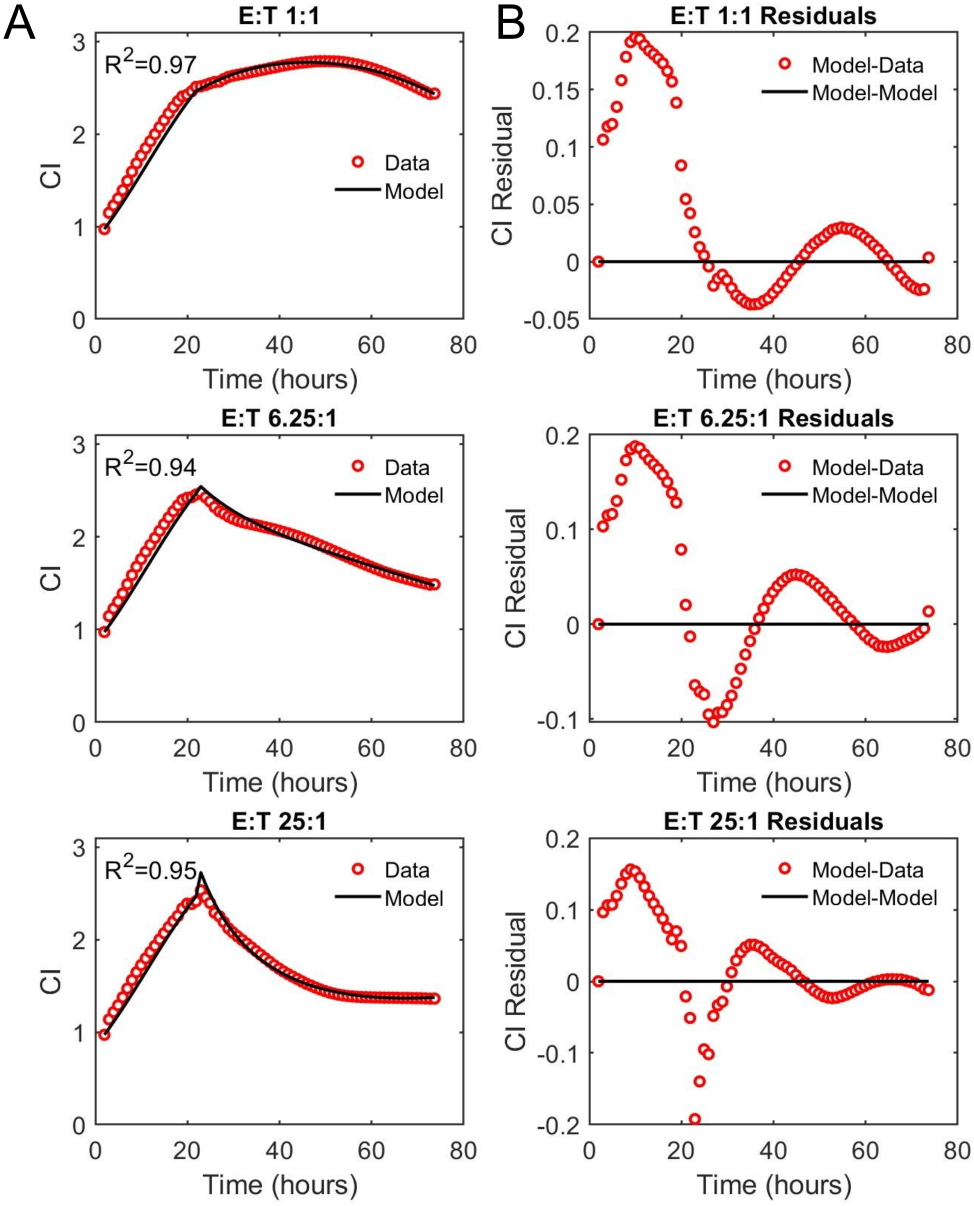

Supplemental Figure 4. Model fit and residuals. (A) Model fit to mean *in vitro* xCELLigence cytotoxicity patient datasets at varying assay initial Effector:Target (E:T) ratios, comparing experimental CI data to estimated values of the model using mean data parameter estimates (**Supplemental Table 4**). (n = 45) **(B)** Residual differences between model simulations and experimental data (orange circles, mean data; black line, model fit).

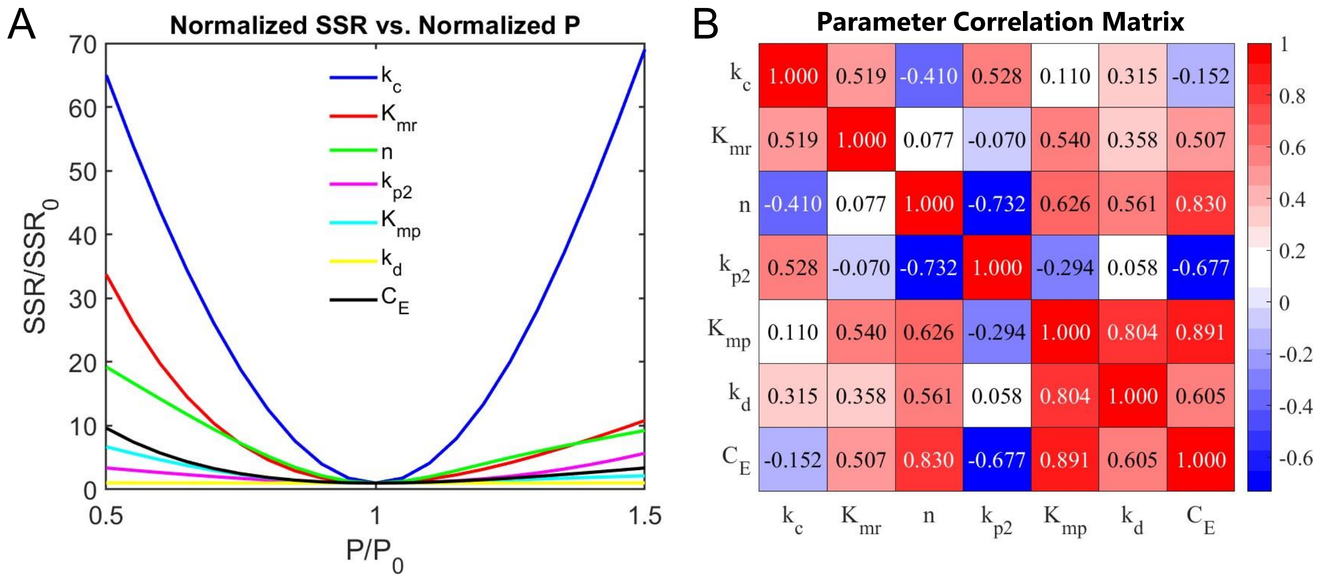

Supplemental Figure 5. Model parameter relationships for mean patient dataset. (A) Model parameter (P) error sensitivity for mean patient data. (SSR, sum of squares of residuals) **(B)** Model parameter correlation matrix calculated using mean data parameter estimates. Analysis for parameters estimated from *in vitro* cytotoxicity model at varying assay initial Effector:Target (E:T) ratios determined using mean data parameter estimates shown in Table 2 and obtained from mean experimental data sets where 1 (red) represents a perfect correlational and 0 (blue) represents no correlation. Parameters defined in **Table 1**.

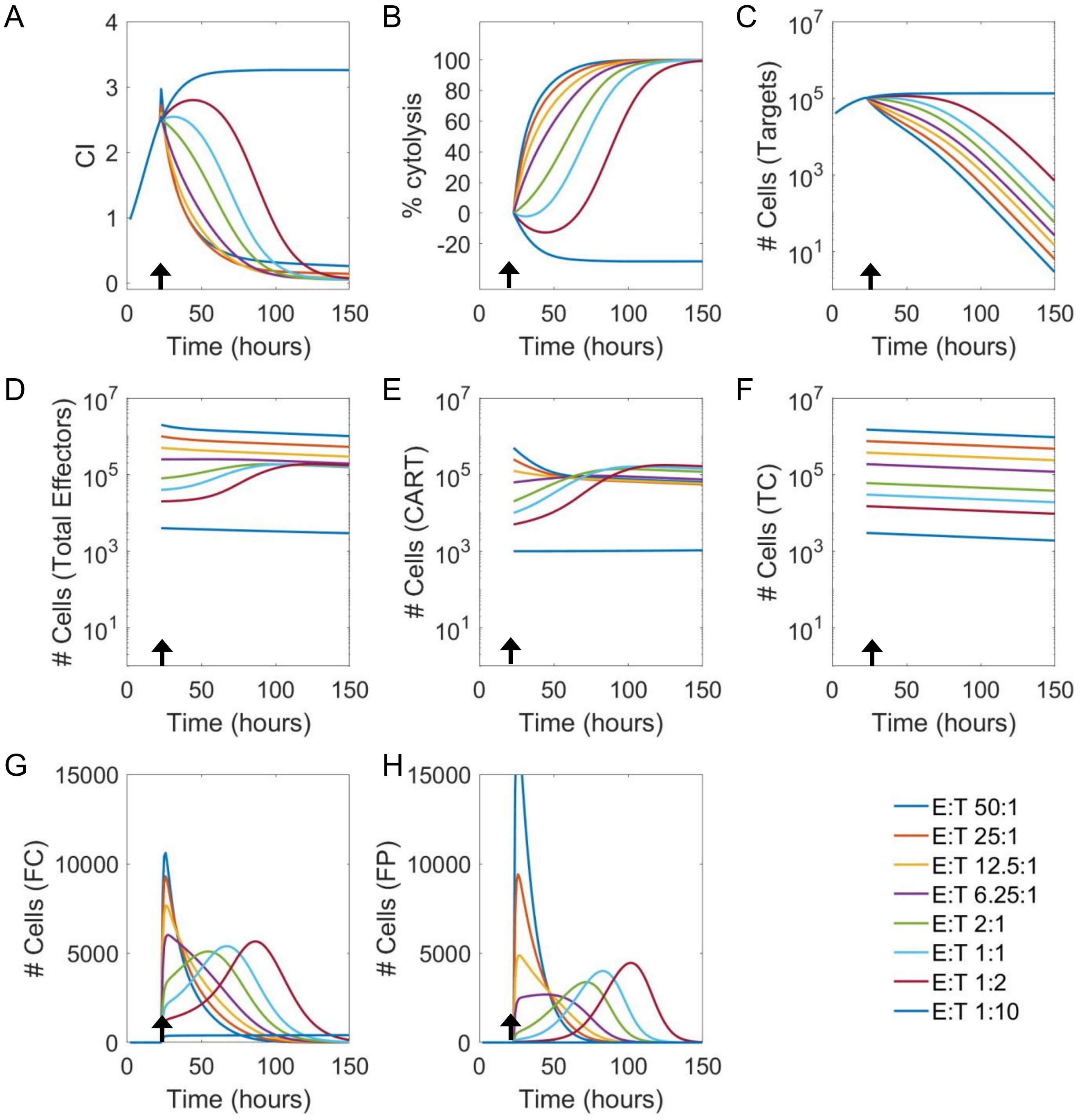

Supplemental Figure 6: Predicted behavior of CAR-T cell-mediated tumor cytolysis in an *in vitro* assay model. Simulations of the *in vitro* cytotoxicity model at varying assay initial Effector:Target (E:T) ratios demonstrates saturation of assay and model cytolysis kinetics at E:T ratios above 12.5:1 and negligible cytolysis at E:T ratio below 1:2. Simulation performed using mean data parameter estimates shown in Table 2 and obtained from experimental data sets. Tumor addition (40,000 cells) at 0 hours. Effector addition at listed E:T ratios at 24 hrs (arrow). **(A)** Predicted impedance (CI). **(B)** Predicted tumor cytolysis. **(C)** Predicted tumor cells, **(D)** Predicted total effectors (CAR-T cells + T-cells (TC)), **(E)** Predicted CAR-T cells, **(F)** Predicted TC, **(G)** Predicted magnitude of the FC function, and **(H)** Predicted magnitude of the FP function.

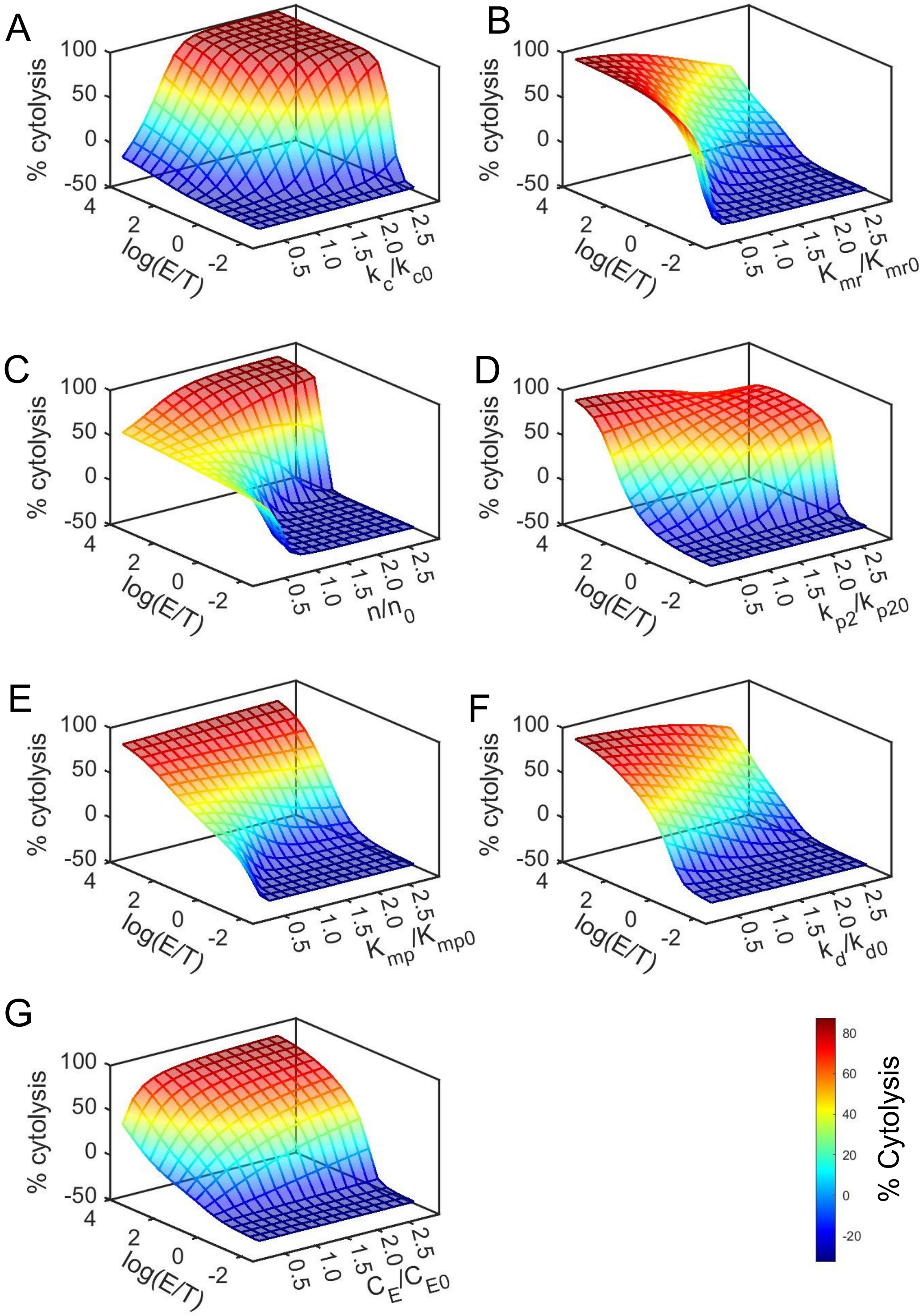

Supplemental Figure 7: Global sensitivity analysis of model showing how varying each parameter changes the % cytolysis predicted by model across a range of E:T ratios. Figure demonstrates how each parameter can vary tumor cytolysis in the *in vitro* assay and how that effect changes with varying assay initial E:T ratios. Paraments were varied between 0.1x estimated values to 3x estimated values is shown on x-axis. E:T ratios varied from 1:10 to 50:1 is shown on y-axis. Predicted cytolysis at 48hrs post effector addition is shown on z-axis. (A) k_c (B) K_{mr} (C) n (D) k_{p2} (E) K_{mp} (F) k_d (G) C_E. Simulations were conducted using mean data parameter estimates.

Supplement Table 2. Clinical patient dataset summary*

Variable	Response Categories (n)
**Early Therapy Response (Day 28)**	R (37)<br>NR (8)
**Late Therapy Response (Day 90) ****	R (35)<br>NR (10)
**Disease Relapse (through day 180)**	No relapse (28)<br>Relapse (9)
**CRS**	Yes (40)<br>No (5)
**Neurotoxicity**	Yes (9)<br>No (36)
**Manufacturing Days**	8 (32)<br>12 (13)
**Disease**	CLL (8)<br>DLBCL (21)<br>FL (5)<br>MCL (11)

*Total n of 45 patient datasets were analyzed in this work from a clinical sample of 56 patient from clinical trial NCT03019055 receiving LV20.19 CAR-T cell therapy with equal initial doses of 2.5e6 CAR-T cells after omitting patients which were not ultimately treated, were missing *in vitro* cytotoxicity assay datasets, or which had datasets where control (tumor only) experiments displayed low, no, or negative tumor growth (defined as CI change < 0.1). Patients with complete responses (CR) or partial responses (PR) were analyzed as responders (R). Patients with progressive disease as non-responders (NR). All DLBCL categories were analyzed together.
** For day 90 response 10 patients with no data collected at day 90 were interpolated response status as follows. CR or PR day 28 and no relapse, data 90 response was considered R (3 patients). Progressive disease at day 28 and relapse, day 90 response was considered NR (7 patients). Other scenarios were not observed in this dataset.
CLL, chronic lymphocytic leukemia; DLBCL, diffuse large B-cell lymphoma; FL, follicular lymphoma; MCL, mantle cell lymphoma.

Supplementary Table 3. P-values for pre-therapy patient sample parameter comparisons using two-sided Wilcoxon rank sum test (results significant at P-value of 0.05 in **bold**)

Parameter	R/NR, Day 28 (n = 45)	R/NR, Day 90 (n = 45)	Relapse Y/N, through day 180 (n = 37)	CRS Y/N (n = 45)	Neurotoxicity Y/N (n = 45)	Manufacturing Days 8/12 (n = 45)
$\mathbf{k_c}$	0.603	0.859	0.058	0.376	0.427	0.531
$\mathbf{K_{mr}}$	0.467	0.859	0.873	0.116	0.876	0.573
**n**	0.667	0.504	0.107	**0.012**	0.809	0.225
$\mathbf{k_{p2}}$	0.801	0.692	0.196	0.240	0.898	0.871
$\mathbf{K_{mp}}$	0.292	0.246	**0.029**	0.255	0.660	0.565
$\mathbf{C_E}$	0.847	0.795	0.250	0.843	0.660	0.310
$\mathbf{k_c}$	0.603	0.859	0.058	0.376	0.427	0.531

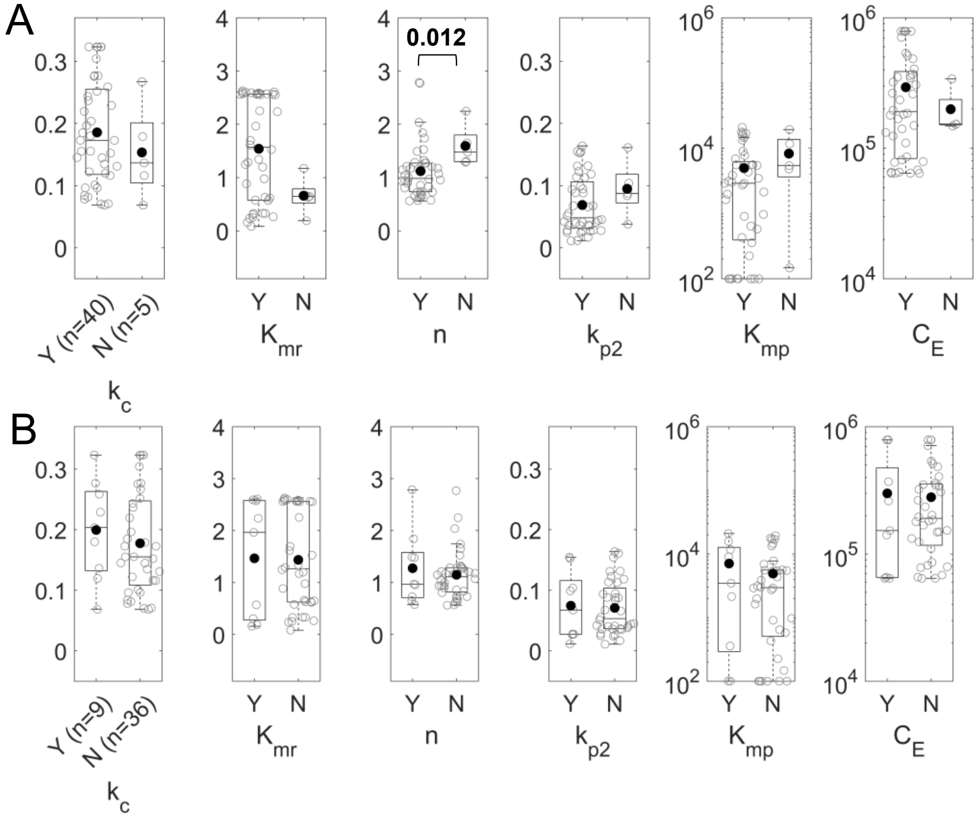

Supplemental Figure 8. Distribution of model parameters estimates between additional patient outcomes of CRS and neurotoxicity. (A) Occurrence of cytokine release syndrome (CRS) (Y) or no CRS (N). **(B)** Occurrence of neurotoxicity (Y) or no neurotoxicity (N). Boxplot with filled circle representing mean, and parameters defined in Table 1. Statistical analysis of differences between parameter values amongst patient groupings was evaluated using Wilcoxon rank sum test (significant P-values as shown, non-significant P-values of greater than 0.05 are not shown , all P-values shown in **Supplementary Table 5**).

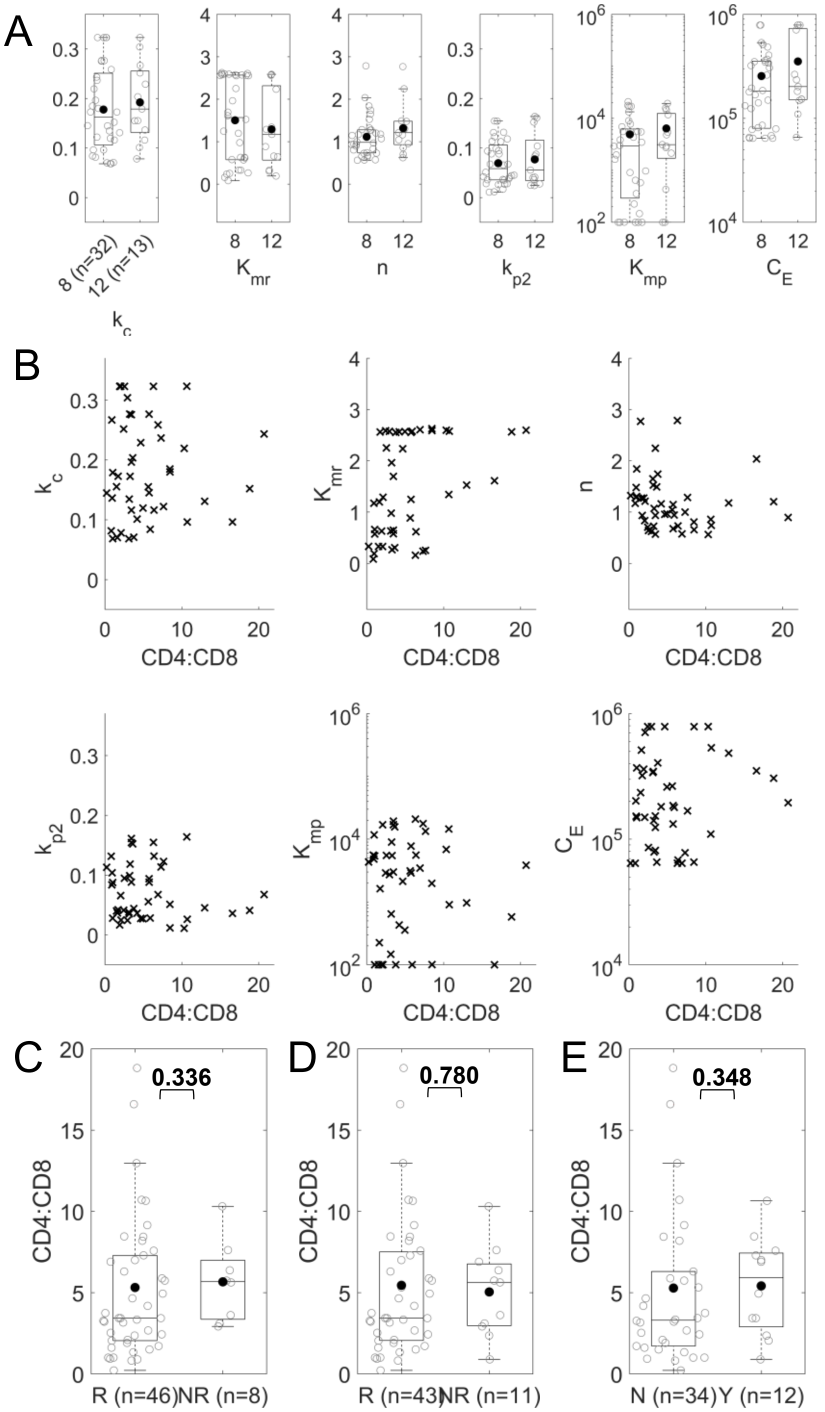

Supplemental Figure 9. Distribution of model parameters by product characteristics. (A) Distribution of product parameters from simulation of individual patient datasets comparing manufacturing protocol harvest at day 8 or 12. Boxplot with filled circle representing mean, and parameters defined in Table 1. (n=45) **(B)** Distribution of parameter values at varying CD4:CD8 ratios observed in all products for individual patients. (n=45) **(C-E)** Distribution of product CD4:CD8 ratios between responders (R) and non-responders (NR) at **(C)** 28 days, **(D)** 90 days, and **(E)** no-relapse (N) and relapse (Y) through 180 days (n=54). Boxplot with filled circle representing mean. Statistical analysis of differences between parameter values amongst patient groupings was evaluated using Wilcoxon rank sum test (CD4:CD8 P-values as shown, all additional P-values shown in **Supplementary Table 5**)

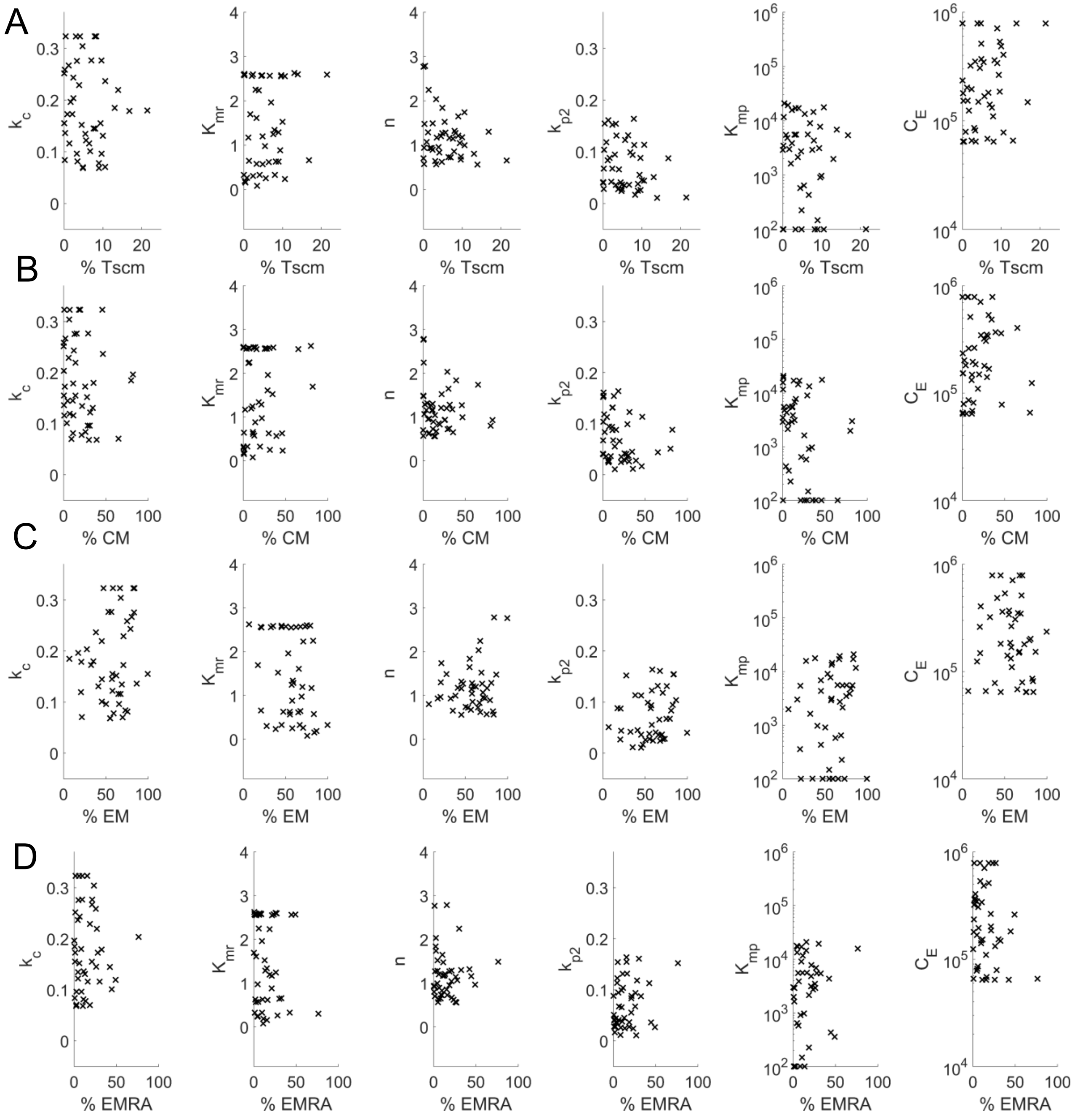

Supplemental Figure 10. Parameter distribution for subpopulations of T cells in products. Distribution of product parameters from simulation of individual patient datasets and separated by percentage of **(A)** Tscm (memory stem cells), **(B)** Tcm (central memory), **(C)** Tem (effector memory), and **(D)** Temra (terminally differentiated). Parameters defined in **Table 1**. (n=45)

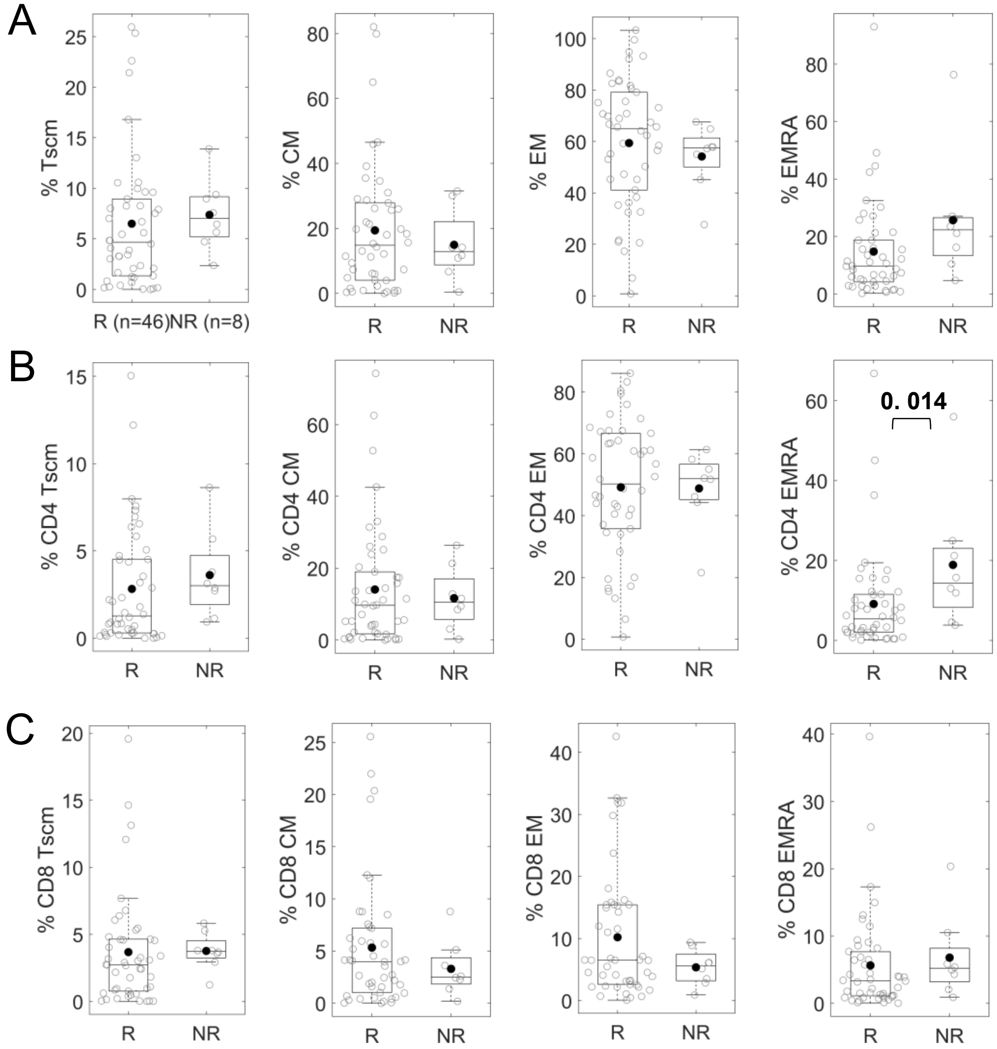

Supplemental Figure 11. Analysis of initial cell populations in CAR-T product between responders and non-responders at day 28. Distribution of product parameters from simulation of individual patient datasets of responders (R) and non-responders (NR) for **(A)** total T cells, **(B)** CD4+ T cells, and **(C)** CD8+ T cells. Populations include memory stem cells (Tscm), central memory (Tcm), effector memory (Tem), terminally differentiated (Temra). Boxplot with filled circle representing mean. Statistical analysis of differences amongst patient groupings was evaluated using two-sided Wilcoxon rank sum test (significant P-values as shown, non-significant P-values of greater than 0.05 not shown, all P-values shown in **Supplementary Table 4**).

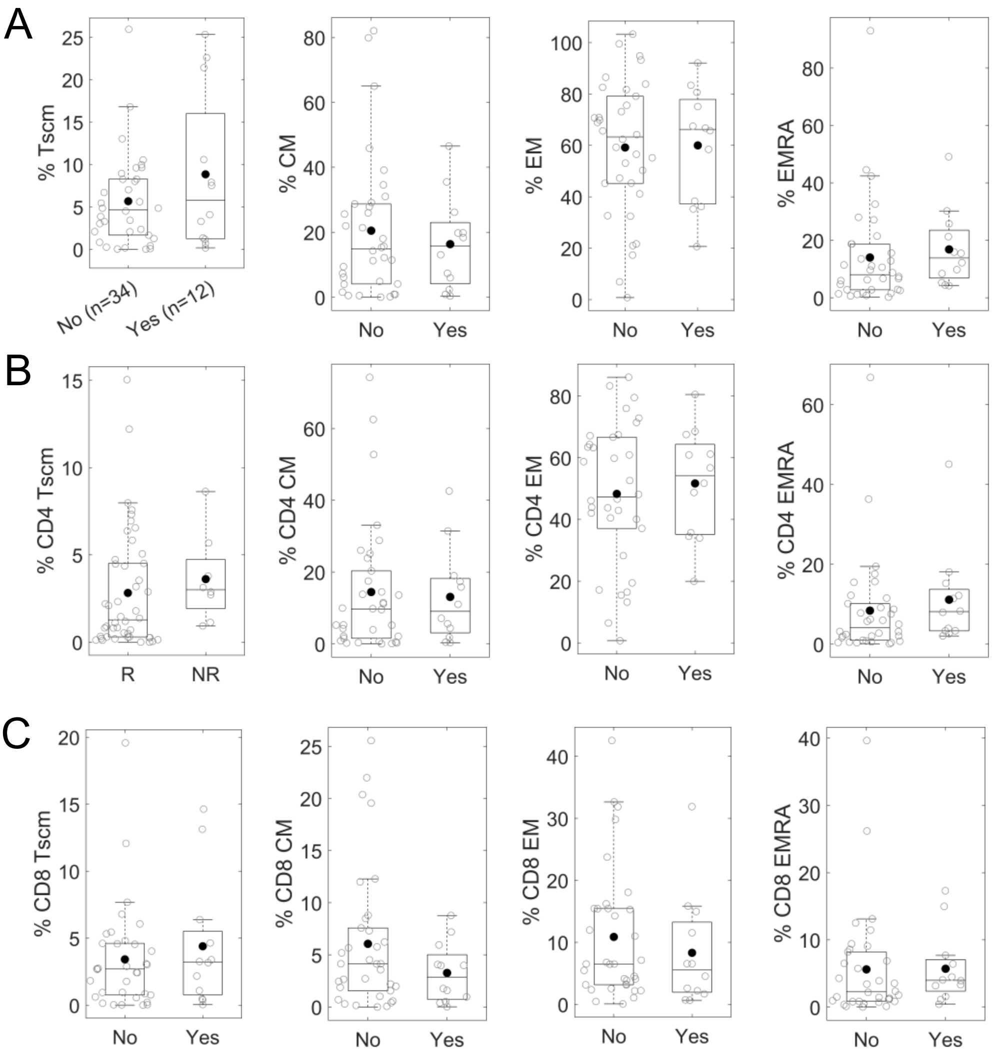

Supplemental Figure 12. Analysis of initial cell populations in CAR-T product between relapse through day 180. Distribution of product parameters from simulation of individual patient datasets of disease relapse through day 180 for **(A)** total T cells, **(B)** CD4+ T cells, and **(C)** CD8+ T cells. Populations include memory stem cells (Tscm), central memory (Tcm), effector memory (Tem), terminally differentiated (Temra). Boxplot with filled circle representing mean. Statistical analysis of differences amongst patient groupings was evaluated using two-sided Wilcoxon rank sum test (no results significant at P-values of greater than 0.05, all P-values shown in **Supplementary Table 4**).

Supplementary Table 4. P-values for pre-therapy patient sample product compositions comparisons using two-sided Wilcoxon rank sum test (results significant at P-value of 0.05 in **bold**)

Comparisons between responders and nonresponses (n = 54)

Cellular Population	Product	CD4	CD8
Tscm	0.228	0.147	0.219
Tcm	0.836	0.855	0.584
Tem	0.312	0.836	0.401
Temra	0.053	**0.014**	0.247

Comparisons between relapse (through day 180) and no relapse (n = 46)

Cellular Population	Product	CD4	CD8
Tscm	0.523	0.255	0.523
Tcm	0.851	0.754	0.244
Tem	1.000	0.736	0.416
Temra	0.173	0.107	0.361

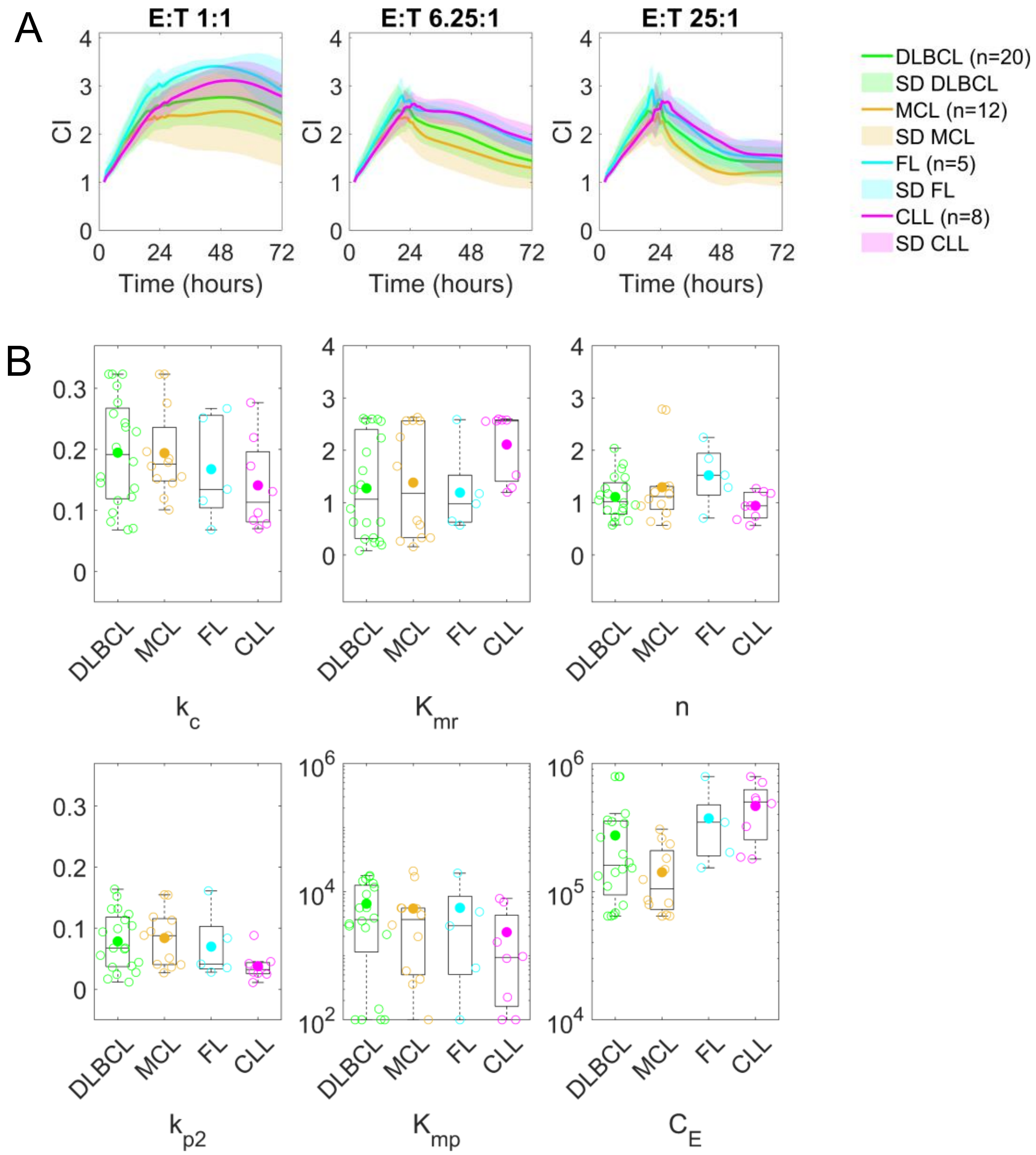

Supplementary Figure 13. In vitro cytotoxicity data and model parameter distribution by patient disease. (A) Analysis of bispecific LV20.19 CAR-T cell cytotoxicity against CD19/20+ Raji cells at various initial Effector (CAR-T cell + T cell):Target (Raji) (E:T) ratios measured by cellular impedance (CI) for various B-cell malignancy types in clinical trial patient populations. This includes diffuse large B-cell lymphoma (DLBCL, green), mantle cell lymphoma (MCL, orange), follicular lymphoma (FL, blue), and chronic lymphocytic leukemia (CLL, pink). **(B)** Distribution of model parameter estimates by B-cell malignancy type. Boxplot with filled circle representing mean, and parameters defined in **Table 1**.

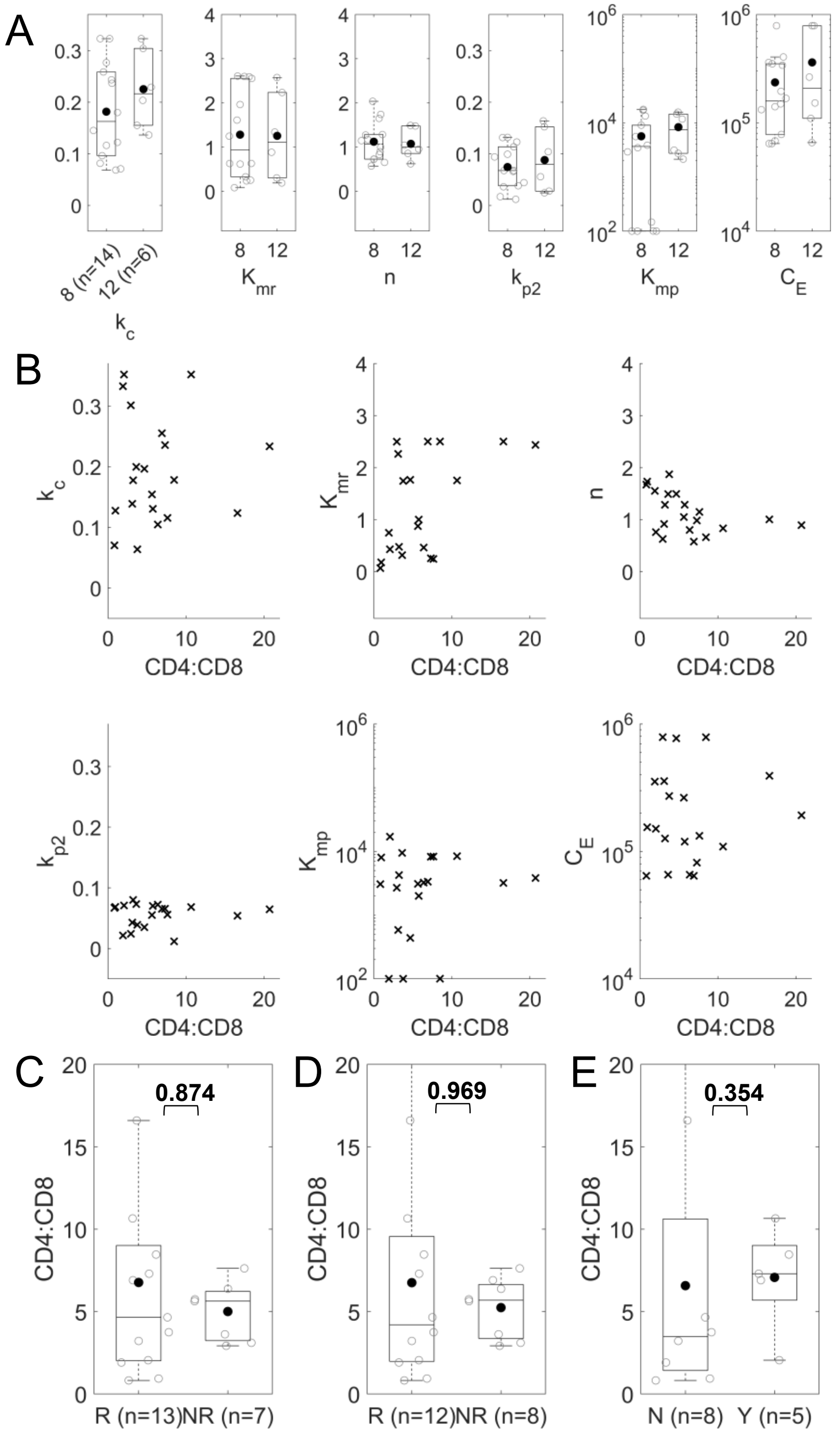

Supplemental Figure 14. Distribution of model parameters by product characteristics (DLBCL only). (A) Distribution of product parameters from simulation of individual patient datasets comparing manufacturing protocol harvest at day 8 or 12. Boxplot with filled circle representing mean, and parameters defined in Table 1. (n=20) **(B)** Distribution of parameter values at varying CD4:CD8 ratios observed in all products for individual patients. (n=20) **(C-E)** Distribution of product CD4:CD8 ratios between responders (R) and non-responders (NR) at **(C)** 28 days, **(D)** 90 days, and **(E)** no-relapse (N) and relapse (Y) at 180 days (n=24). Boxplot with filled circle representing mean. Statistical analysis of differences between parameter values amongst patient groupings was evaluated using Wilcoxon rank sum test (CD4:CD8 P-values as shown, all additional P-values shown in **Supplementary Table 5**)

Supplementary Table 5. P-values for pre-therapy patient sample parameter comparisons using two-sided Wilcoxon rank sum test (results significant at P-value of 0.05 in **bold**, DLBCL only)

Parameter	R/NR, Day 28 (n = 45)	R/NR, Day 90 (n = 45)	Relapse Y/N, through day 180 (n = 37)	CRS Y/N (n = 45)	Neurotoxicity Y/N (n = 45)	Manufacturing Days 8/12 (n = 45)
$\mathbf{k_c}$	0.204	0.374	0.168	0.115	0.536	0.342
$\mathbf{K_{mr}}$	0.428	0.969	0.833	0.231	0.386	0.902
**n**	0.634	0.847	**0.030**	0.115	0.303	0.967
$\mathbf{k_{p2}}$	0.526	0.563	0.724	0.950	0.902	0.711
$\mathbf{K_{mp}}$	0.874	0.908	0.127	0.950	0.773	0.483
$\mathbf{C_E}$	0.874	0.418	0.354	0.753	0.902	0.650
$\mathbf{k_d}$	0.204	0.374	0.168	0.115	0.536	0.342

Supplementary Table 6. Study results for varying CAR-T product CD4:CD8 ratios including ratios utilized, CAR-T product transduction efficiencies (TE) for each ratio, resulting model parameter estimates, and model goodness for fit for each cytotoxicity dataset.

	Study r0101	Study r0401	Study r1001	Study rCD4
**CD4:CD8 ratio**	1.0	4.0	10.0	CD4 only
**CD4:CD8 ratio (CAR+ cells)**	1.5	6.0	15.0	CD4 only
**CAR+ % (CD4 cells)**	0.161	0.161	0.161	0.161
**CAR+ % (CD8 cells)**	0.107	0.107	0.107	0.107
**TE**	0.134	0.150	0.156	0.161
$\mathbf{k_c}$	0.153	0.238	0.156	0.300
$\mathbf{K_{mr}}$	1.504	1.016	0.919	0.846
**n**	0.533	1.601	1.016	3.999
$\mathbf{k_{p2}}$	0.120	0.077	0.057	0.058
$\mathbf{K_{mp}}$	6.23E+03	4.65E+03	2.65E+03	2.71E+03
$\mathbf{C_E}$	2.57E+05	3.38E+05	4.20E+05	4.44E+05
$\mathbf{k_d}$	0.004	0.000	0.000	0.000
**R^2 (ET 1:1)**	0.940	0.960	0.970	0.970
**R^2 (E:T 6.25:1)**	0.960	0.960	0.960	0.970
**R^2 (E:T 25:1)**	0.970	0.990	0.990	0.990

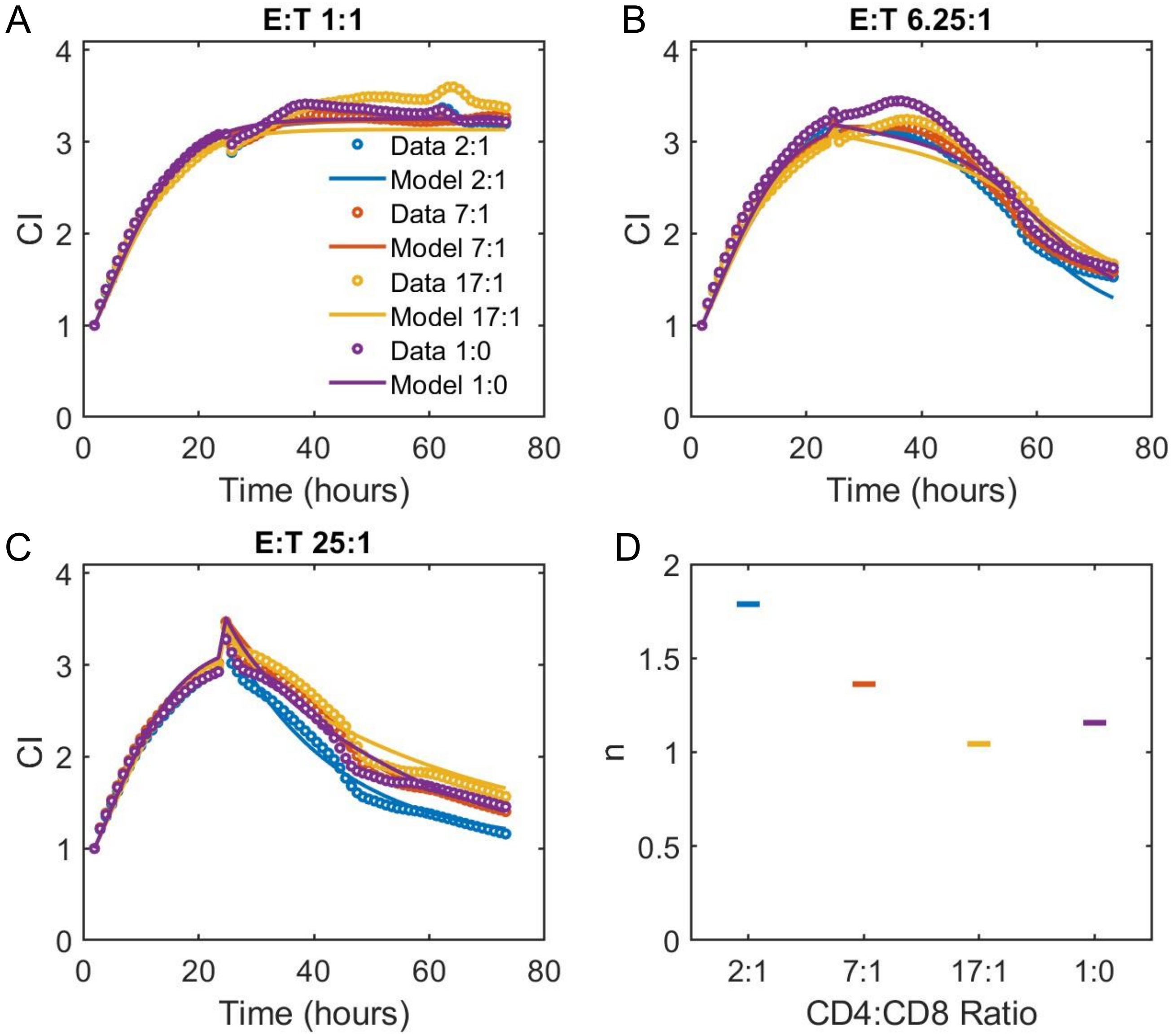

Supplemental Figure 15. Cytotoxicity data and model simulations demonstrating how changes in CD4:CD8 ratios of healthily PBCM donor derived frozen/thawed CAR-T product alters parameter n. Analysis of bispecific LV20.19 CAR-T cell cytotoxicity against CD19/20+ Raji cells using CAR-T product manufactured with healthy PBMC donor and adjusted to fixed 1:1, 4:1, 10:1 and 1:0 (CD4 only) CD4:CD8 ratios among CD3+ cells which corresponded to 1:5:1, 6:1, 15:1, and 1:0 (CD4 only) CD4:CD8 ratios (as denoted in legend) among CAR+ CD3+ cells respectively at various initial Effector (CAR-T cell + T cell):Target (Raji) (E:T) ratios measured by cellular impedance (CI) over time for **(A)** E:T 1:1, **(B)** E:T 6.25:1, and **(C)** E:T 25:1. **(D)** Predicted value for model parameter n at various CAR-T product CD4:CD8 ratios. Transduction efficiencies, model parameter estimates, and model goodness of fit shown in **Supplementary Table 7**. This data represents a replicate of experiment in **Figure 6** with thawed CAR-T cells.

Supplementary Table 7. Study results for varying CAR-T product CD4:CD8 ratios including ratios utilized, CAR-T product transduction efficiencies (TE) for each ratio, resulting model parameter estimates, and model goodness for fit for each cytotoxicity dataset. Data represents replicated of Supplementary Figure 6 with thawed CAR-T cells.

	Study r0101	Study r0401	Study r1001	Study rCD4
**CD4:CD8 ratio**	1.0	4.0	10.0	CD4 only
**CD4:CD8 ratio (CAR+ cells)**	2.0	7.0	17.0	CD4 only
**CAR+ % (CD4 cells)**	0.148	0.148	0.148	0.148
**CAR+ % (CD8 cells)**	0.086	0.086	0.086	0.086
**TE**	0.125	0.136	0.142	0.148
$\mathbf{k_c}$	0.107	0.127	0.132	0.134
$\mathbf{K_{mr}}$	1.787	1.362	1.045	1.155
**n**	1.442	3.000	3.000	3.000
$\mathbf{k_{p2}}$	0.040	0.101	0.050	0.057
$\mathbf{K_{mp}}$	1.01E+03	1.77E+03	2.00E+03	2.00E+03
$\mathbf{C_E}$	1.99E+06	2.00E+06	2.00E+06	2.00E+06
$\mathbf{k_d}$	0.000	0.000	0.000	0.000
$\mathbf{R^2}$ **(ET 1:1)**	0.98	0.98	0.96	0.96
$\mathbf{R^2}$ **(E:T 6.25:1)**	0.98	0.99	0.92	0.91
$\mathbf{R^2}$ **(E:T 25:1)**	0.98	0.99	0.85	0.98
